# Neuroprotective role of sialic-acid-binding immunoglobulin-like lectin-11 in humanized transgenic mice

**DOI:** 10.3389/fnins.2024.1504765

**Published:** 2024-12-23

**Authors:** Tawfik Abou Assale, Negin Afrang, Jannis Wissfeld, German Cuevas-Rios, Christine Klaus, Bettina Linnartz-Gerlach, Harald Neumann

**Affiliations:** Institute of Reconstructive Neurobiology, Medical Faculty and University Hospital of Bonn, University of Bonn, Bonn, Germany

**Keywords:** Siglec-11, aging, microglia, neurodegeneration, neuroinflammation, oxidative stress

## Abstract

Brain aging is a chronic process linked to inflammation, microglial activation, and oxidative damage, which can ultimately lead to neuronal loss. Sialic acid-binding immunoglobulin-like lectin-11 (SIGLEC-11) is a human lineage-specific microglial cell surface receptor that recognizes *α*-2-8-linked oligo−/polysialylated glycomolecules with inhibitory effects on the microglial inflammatory pathways. Recently, the *SIGLEC11* gene locus was prioritized as a top tier microglial gene with potential causality to Alzheimer’s disease, although its role in inflammation and neurodegeneration remains poorly understood. In this study, aged Siglec-11 transgenic (tg) mice, which expressed the human SIGLEC-11 receptor on microglia and tissue macrophages, were investigated. The brains of the Siglec-11 tg mice were analyzed in 6-month-old mature mice and 24-month-old aged mice using immunohistochemistry and transcriptomics. Results showed decreased density and fewer clusters of ionized calcium binding adaptor molecule 1 (Iba1)-positive microglial cells in the hippocampus and substantia nigra, as well as less lipid-laden microglia in the Siglec-11 tg in comparison to wildtype (WT) controls. Additionally, Siglec-11 tg mice exhibited less age-related neuronal loss in the substantia nigra *pars compacta* in comparison to WT mice. Transcriptome analysis revealed suppression of oxidative phosphorylation and inflammatory pathways in Siglec-11 tg brains at 6 months, with further suppression of complement and coagulation cascades at 24 months of age in comparison to WT mice. Gene transcript levels of the pro-inflammatory cytokines *tumor necrosis factor alpha* (*Tnf*) and *interleukin 1 beta* (*Il-1β*) as well as the oxidative stress markers *cytochrome b-245 alpha* and *beta* (*Cyba* and *Cybb*) and the nitric oxide synthase 2 (*Nos2*), were reduced in the brains of 24-month-old Siglec-11 tg mice relative to WT controls. Brains of 24-month-old Siglec-11 tg mice also exhibited lower gene transcription of complement components 3, 4, and integrin alpha M (*C3, C4,* and *Itgam*), along with the complement C1q subcomponents a-c (*C1qa, C1qb*, and *C1qc*). In summary, aged Siglec-11 tg mice displayed reduced brain inflammation and oxidative stress, as well as protection against age-related neuronal loss in the substantia nigra.

## Introduction

Brain aging is a complex process that affects various functions and often leads to cognitive decline (Rodrigue and Kennedy, 2011). Common changes in the aging brain include neurodegeneration, accumulation of lipid-laden microglia, impaired synaptic neurotransmission, and a buildup of oxidative damage-related molecules (Lee and Kim, 2022). Microglia, the resident innate immune cells in the central nervous system (CNS), continuously monitor the microenvironment, detecting misfolded protein plaques, damaged or apoptotic cells, and other injured or degenerated materials. Chronic reactive microgliosis and the resulting neuroinflammation have been implicated in age-related neurodegeneration, which can lead to dementia (Luo et al., 2010).

Variants of several SIGLEC genes have been associated with either increased risk or protection against the development of Alzheimer’s disease (AD) and dementia (Wißfeld et al., 2024). In addition to the *SIGLEC3/CD33* gene, the *SIGLEC11* gene locus has been identified as a key microglial gene with potential links to AD risk (Bellenguez et al., 2022). Bellenguez et al. (2022) prioritized AD/dementia-associated genes based on their relevance to disease processes, finding *SIGLEC11* to be the most significant microglial gene linked to AD.

Sialic acid-binding immunoglobulin-type lectins (SIGLECs) are recognition receptors that bind sialylated glycoproteins and glycolipids, primarily expressed on immune cells (Macauley et al., 2014). Most CD33-related (CD33r) SIGLECs, including SIGLEC-11, contain one or more immunoreceptor tyrosine-based inhibition motifs (ITIMs) in the cytoplasmic domain. When SIGLEC receptors bind to their sialylated ligands, they typically inhibit signals from immunoreceptor tyrosine-based activation motif (ITAM) receptors, leading to the downregulation of pro-inflammatory immune responses and phagocytosis in microglia and tissue macrophages (Klaus et al., 2021; Wang and Neumann, 2010).

Interestingly, SIGLEC-11 has been identified as a “human-specific microglial receptor,” as it is not expressed on the microglia of non-human primates and is almost completely absent in rodents (Angata et al., 2002; Hayakawa et al., 2005; Khan et al., 2020; Wang et al., 2011, 2012). Previous *in vitro* studies showed that SIGLEC-11 preferentially binds to α2-8-linked sialic acid oligomers (oligoSia) and polymers (polySia) (Angata et al., 2002; Hayakawa et al., 2005; Shahraz et al., 2015). Furthermore, SIGLEC-11 reduces microglial neurotoxicity in culture by interacting with the intact oligo-/polysialylated glycocalyx of neighboring cells (Wang and Neumann, 2010).

In this study, we analyzed the effect of SIGLEC-11 on normal aging in 6-month-old mature and 24-month-old aged humanized Siglec-11 transgenic (tg) mice, which express the human SIGLEC-11 receptor on microglia and tissue macrophages (Karlstetter et al., 2017; Liao et al., 2021). We found that aged 24-month-old Siglec-11 tg mice exhibited reduced microglial density, fewer microglial clusters, and fewer lipid-laden microglia compared to the wildtype (WT) control mice. Additionally, Siglec-11 tg mice experienced less age-related neuronal loss in the substantia nigra at 24 months compared to WT mice. Furthermore, aged Siglec-11 tg mice demonstrated suppression of inflammatory, oxidative stress, and apoptotic pathways relative to WT mice.

Thus, SIGLEC-11 exerted anti-inflammatory effects that were neuroprotective in the aged brains of Siglec-11 tg mice.

## Materials and methods

### Animals and tissue collection

All animal experiments have been approved by the authors’ institutional and governmental review boards and have complied with the Helsinki Declaration. C57BL/6J mice were obtained from Charles River. Siglec-11 transgenic (tg) mice (SIGLEC-11+/−) were generated by pro-nuclear injection of a macrophage/microglial-specific Iba1 mini-promotor as previously described (Karlstetter et al., 2017; Liao et al., 2021) and were backcrossed for at least 10 generations with C57BL/6J mice before performing experiments. REDExtract-N-Amp Tissue polymerase chain reaction (PCR) Kit (Sigma) was used to perform SIGLEC11 genotyping PCR for the Siglec-11 tg mice. All mice were maintained in specific pathogen free environment with free access to both water and food. In this study, we analyzed 1.5-, 6-, and 24-month-old male mice as juvenile, mature, and aged groups, respectively. We only investigated male mice, as their substantia nigra dopaminergic neurons are more susceptible to the effects of aging in comparison to female mice (Howell et al., 2020). For tissue collection, mice were euthanized and then transcardially perfused with ice-cold phosphate buffer saline (PBS). Brains were collected and cut into right and left hemispheres for immunohistochemistry and RNA isolation, respectively.

### RNA sequencing, gene transcript pathway, and sqRT-PCR analyses

Left brain hemispheres of mice were collected as described above and homogenized in 1 mL QIAzol Lysis reagent (Qiagen, Germany) for 6 min at 50 Hz using a Tissue Lyser LT (Qiagen, Germany) and stainless-steel beads (Qiagen, Germany). Total RNA was extracted using the spin column protocol of the RNeasy^®^ Mini Kit (Qiagen, Germany) according to the manufacturer’s protocol. The RNA concentration was measured using a Nanodrop system (NanoDrop 200c, Thermo Fisher Scientific, Waltham, MA) and diluted to 100 ng/μl. RNA integrity was assessed using 2,100 Bioanalyzer (Agilent, Santa Clara, CA). For transcriptome analysis, library preparation (QuantSeq 3′ mRNA-Seq Library Prep Kit, Lexogen) with an input of 100 ng total RNA, quality control (Tapestation 2,200, Fa. Agilent), and RNA sequencing were performed at the Next Generation Sequencing (NGS) Core Facility (Medical Faculty, University Hospital of Bonn) with 2 × 10^7^ single-end reads per sample on a NovaSeq 6,000 (Illumina). After performing quality control on raw reads using FastQC (v0.11.8) and MultiQC (v1.7), adapters were trimmed from the reads using Bbmerge/BBDUK (Bushnell et al., 2017). Reads were aligned to the mouse reference genome mm10 (GRCm38) with the ensemble gene annotation version 101 using STAR [v2.7.3a] (Dobin et al., 2013) with standard parameters. Read count generation was performed using featureCounts/Subread [v2.0.0] (Liao et al., 2014) ignoring multimapping reads. Then, differential expression analysis was performed with R [v4.3.1] in RStudio [v2023.6.2, build 531] (RStudio Team, 2020) using DESeq2 [v1.42.0] (Love et al., 2014). Transcript annotations were retrieved using the Bioconductor package org.Mm.eg.db [v3.11.4] (Carlson, 2019), and plots were created using ggplot2 [v3.3.3] (Wickham, 2016). Pathway enrichment analyses were performed using clusterProfiler [v3.16.1] (Yu et al., 2012) and GSEA Desktop[v4.2.2] (Mootha et al., 2003; Subramanian et al., 2005) with log2FC ≥ 1 and adjusted *p*-value <0.05. Gene transcripts of each group within each age were contrasted and used for the differential analysis to extract the differentially expressed genes, enriched pathways, and hallmark gene sets. For reverse transcription (RT) of isolated RNA, Superscript® III Reverse Transcriptase (Invitrogen, Germany) and random hexamer oligonucleotides (Roche, Germany) were used following the manufacturers’ protocol for SuperScript First-Strand Synthesis (Invitrogen, Germany). The cDNA concentration was measured with a Nanodrop system (NanoDrop 200c, Thermo Fisher Scientific). Semi-quantitative real-time polymerase chain reaction (sqRT-PCR) with specific oligonucleotides (for sequences see Supplementary Table S1) was performed with SYBR Green PCR Master Mix (Invitrogen, Germany) using the Eppendorf epigradient S Mastercycler^®^ (Eppendorf, Wesseling-Berzdorf, Germany). Amplification specificity was confirmed by the analysis of the melting curves. Transcripts of the *glyceraldehyde-3-phosphate dehydrogenase* (*GAPDH*) housekeeping gene were used as internal controls. For sqRT-PCR gene transcription quantification, the values were adjusted relative to their corresponding *GAPDH* levels and assessed using the delta–delta CT approach. Initially, the average CT value for *GAPDH* was determined for each animal and deducted from the average CT value of each gene primer, resulting in the delta CT value. Subsequently, the mean delta CT value was calculated for the 6-month-old WT control group. The delta delta-CT value for each gene primer per animal was derived by subtracting the mean delta CT value of the control group from the delta CT value of the respective primer. The power of the negative delta delta-CT value for each gene primer per animal was then calculated and utilized as the relative measurement of gene transcription.

### Immunohistochemistry

The right brain hemispheres were collected as described above and immersed in 4% paraformaldehyde (PFA, Sigma, Germany) for 24 h, followed by 30% sucrose (Sigma, Germany) supplemented with 0.1% sodium azide (Sigma, Germany) at 4°C until processed further. The hemispheres were embedded in O.C.T.™ Compound, Tissue Tek^®^ (Sakura, Torrance, CA). Then, brains were sectioned in the rostral-caudal coronal plane with a thickness of 20 μm and mounted onto superfrost slides and stored at −20°C before staining. For SIGLEC-11 receptor staining, slides containing substantia nigra *pars reticulata* (SN*pr*) were blocked with PBS containing 10% bovine serum albumin (BSA; Roth), 0.2% triton X-100 (Sigma-Aldrich/Merck) and 5% normal goat serum (NGS; Sigma-Aldrich/Merck) for 2 h at room temperature (RT), followed by overnight incubation with anti-SIGLEC11 antibody (mouse anti-Siglec-11, clone 4C4, 1:100, BioLegend, #681702) and antibodies against the microglial marker ionized calcium binding adaptor molecule 1 (Iba1, 1:500, rabbit-anti, Synaptic Systems #234003) at 4°C. After three times washing with PBS, the slices were incubated with the corresponding secondary antibodies in blocking solution for 2 h at RT (Cy3-conjugated goat-anti-mouse IgG F[ab′]2 antibody, 1:200, Dianova #115–166-072 and Alexa-488-conjugated goat-anti-rabbit antibody, 1:400, Invitrogen #A11008). For neuronal quantifications, slides containing SN *pars compacta* (SN*pc*) matched levels (e.g., Bregma −3.20, −3.40 and −3.80 mm) per animal were blocked with blocking solution for 1 h followed by 2 h incubation with primary antibodies against the dopaminergic neuron marker tyrosine hydroxylase (TH; 1:500, rabbit-anti, Sigma-Aldrich/Merck #T8700-1VL) and against neuronal nuclei marker NeuN (1:500, mouse-anti-human/mouse, Millipore #MAB377) in blocking solution. After washing three times with PBS, the slices were incubated with the corresponding secondary antibodies in blocking solution for 2 h at RT (Alexa-488-conjugated goat-anti-rabbit antibody, 1:500, Invitrogen #A11008 and Cy3-conjugated goat anti-mouse IgG F[ab′]2 antibody, 1:200, Dianova #115–166-072). For microglia evaluation, sections at Bregma level −3.18 mm were blocked with blocking solution (PBS containing 10% BSA, 0.25% triton X-100) for 2 h at RT followed by overnight incubation at 4°C with primary antibodies against microglial marker ionized calcium binding adaptor molecule1 (Iba1, 1:500, rabbit-anti, Synaptic Systems #234003) and lysosomal marker Cluster of Differentiation 68 (CD68, 1:500, rat-anti-mouse, BioRad #MCA1957) in incubation solution (PBS containing 5% BSA and 0.05% triton X-100). After five times washing with 1× PBS, the slices were incubated with corresponding secondary antibodies in blocking solution for 2 h at RT (Alexa-488-conjugated goat-anti-rabbit antibody, 1:400, Invitrogen #A11008 and Cy3-conjugated goat-anti-rat IgG F[ab′]2 antibody, 1:200, Dianova #112–166-072). For lipid-droplet accumulation in Iba1+ cells analysis, sections were blocked and permeabilized using 10% BSA and 0.25% TritonX-100 in PBS followed by the primary antibody rabbit-anti-ionized calcium-binding adapter molecule 1 (Iba1, 1:500; Wako, Japan) in incubation solution (IS; 5% BSA and 0.05% TritonX-100 in PBS) overnight at 4°C. After three washing steps in IS, the sections were incubated in the corresponding Alexa-488-coupled secondary antibodies (1:400, Jackson ImmunoResearch Laboratories, United Kingdom) in IS for 2 h at RT. All stainings were finalized by washing twice with PBS, staining with 4′,6-diamidino-2-phenylindole (DAPI, 1:10,000) for 30 s, followed by washing once with PBS. Finally, the slides were mounted with Aqua-poly/mount (Polysciences) and stored at 4°C until images were taken.

### Microglial density, activation, and clustering analysis

To assess the microglial activation status, five z-stacks of Iba1 stained hippocampus and substantia nigra *pars reticulata* (SN*pr*) were acquired with an SP8 confocal microscope using a 40× objective lens and the LAS-X software (Leica, Germany). All number-coded images were analyzed by a blinded investigator, and the levels were defined according to the mouse brain atlas of Paxinos and Franklin. Images were exported as TIFF files and analyzed with ImageJ software (National Institute of Health). Microglial density was quantified by counting Iba1-/DAPI-double-positive cells per area in each image and calculating the average for each mouse. For microglial cluster analysis, three or more Iba1 positive cells in close proximity to each other (found within a 50 μm radius) were defined as a microglial cell cluster. The clusters were then counted in each image, and the average for each mouse was calculated. To measure the CD68 intensity per soma, the soma area of five Iba1/CD68-positive cells in each image was selected. CD68 fluorescence intensity was measured in each soma, and the background fluorescence intensity was substracted. The mean measured CD68 fluorescence intensity for the five somas was calculated per animal, and the mean for each group was calculated.

### Neuronal density quantification

To quantify the neurons, TH- and NeuN-stained cells in the substantia nigra *pars compacta* (SN*pc*) or only NeuN-positive cells in the Cornu Ammonis 3 (CA3) area of the hippocampus were acquired and analyzed by a blinded investigator with an AxioObserver Z1 inverted microscope with Apotome (Carl Zeiss) via AxioCam Rm (AxioVision software) as described before (Bodea et al., 2014; Shahraz et al., 2021). Using ImageJ software (National Institute of Health), TH- and NeuN-positive cells were counted, and the area was measured. Neuronal density was calculated as number of counted cells per area, and the mean for each mouse was taken.

### Lipid-laden microglia quantification

Images of Iba1-stained hippocampal and SN*pr* were acquired with an SP8 confocal microscope using a 40× objective lens and the LAS-X software (Leica, Germany). The autofluorescence emission of lipid droplets was imaged using the same detection settings as the Cy3 dye emission spectrum. All number-coded images were analyzed by a blinded investigator using the ImageJ software. The levels were defined according to the mouse brain atlas of Paxinos and Franklin. For microglial lipid accumulation analysis, z-stacks were taken at the Bregma level −2.7 to −2.8 mm. Iba1+ cells were quantified in each image and divided by the measured image area to calculate the number of cells per area. The mean number of Iba1+ cells/area was then calculated for each group. Next, lipid droplets- and Iba1-double-positive cells were also counted, and their ratio to total Iba1+ number was calculated. The ratio was then divided by the imaged area, and the mean for each group was calculated.

### Statistical analysis

Data are presented as mean ± SEM (if not stated otherwise). The mean from at least three mice was collected and analyzed, and the n-number represents the number of investigated mice. Results were normalized to the mean of the adult (6-month-old) WT control mice. Outliers were identified with Grubbs’ test and excluded. Data for 6- and 24-month-old age groups were analyzed with two-way analysis of variance (ANOVA) followed by Fisher’s Least Significance Variance (LSD) *post hoc* test. For the 1.5-month-old age group, data were tested for normality using Shapiro–Wilk test and analyzed with Student’s *t*-test. Statistical analysis was carried out using GraphPad Prism (v9.0.0). Only statistically significant comparisons were depicted in the figures as *p ≤ 0.05, **p ≤ 0.01, ***p ≤ 0.001.

## Results

### Decreased microglial density and less microglial clusters in aged Siglec-11 tg mice

**Figure 1:**
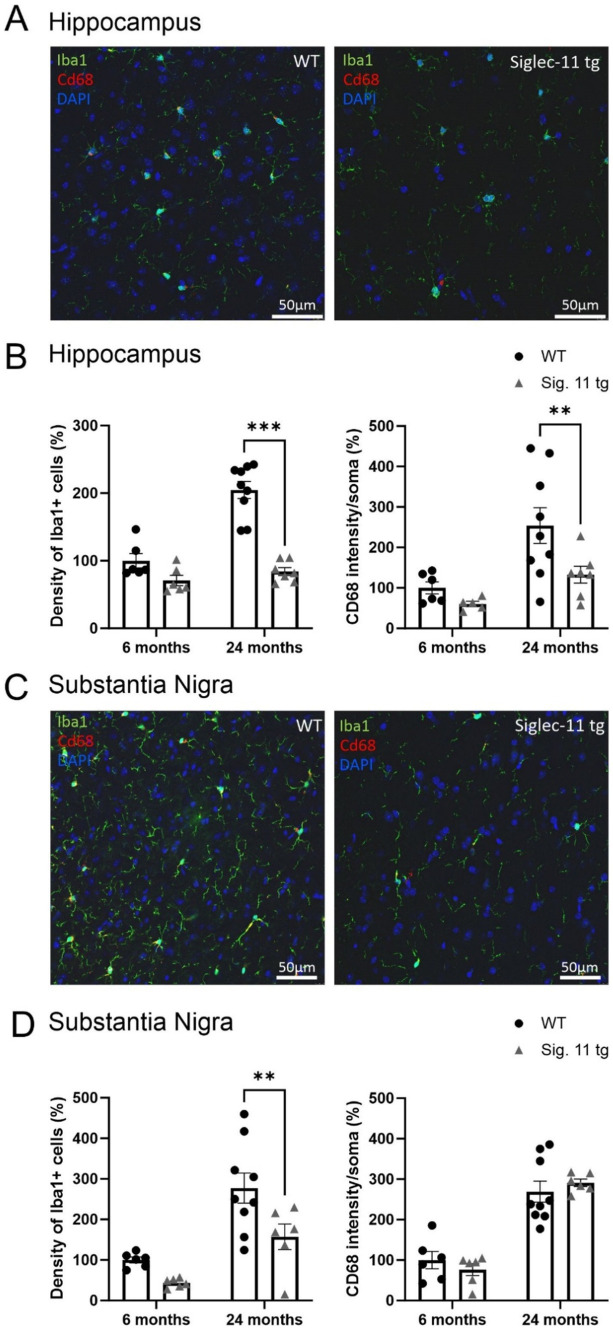
Less microglial density in aged Siglec-11 transgenic compared to wildtype mice. Hippocampal and substantia nigra tissue was co-stained with antibodies directed against the microglial transmembrane protein Iba1 and the phagolysosomal protein CD68. **(A)** Representative images of Iba1 (green), Cd68 (red) and DAPI (blue) in the hippocampus of 24-month-old wildtype (WT) control and Siglec-11 transgenic (tg) mice are shown. **(B)** Quantification of hippocampal Iba1 cell density showed decreased levels at 24 months of age in Siglec-11 tg mice compared to WT mice. In addition, intensity of CD68 in the soma of microglia was decreased in 24-month-old Siglec-11 tg compared to the WT mice. **(C)** Representative images of Iba1 (green), Cd68 (red), and DAPI (blue) in the substantia nigra *pars reticulata* of 24-month-old WT and Siglec-11 tg mice are shown. **(D)** Quantification in the substantia nigra *pars reticulata* showed decreased Iba1 cell density in Siglec-11 tg mice at 24 months of age compared to WT mice. Data were analyzed with two-way ANOVA and Fisher’s LSD *post hoc* test and are shown as mean ± SEM. *n* = 5–9 mice per group. Scale bars 50 μm. **p ≤ 0.01, ***p ≤ 0.001.

Humanized Siglec-11 transgenic (tg) mice, that express the human SIGLEC-11 receptor on microglia and macrophages (Karlstetter et al., 2017; Liao et al., 2021), were used, since expression of SIGLEC-11 on microglia is human-lineage specific. First, we confirmed expression of Siglec-11 in the brain of this transgenic mouse line (Supplementary Figure S1). Immunohistochemistry showed expression of SIGLEC-11 on microglia in the brains of Siglec-11 tg mice co-stained with antibodies against the microglial marker protein ionized calcium-binding adaptor molecule 1 (Iba1). We analyzed microglial density, microglial expression of the phagolysosomal protein CD68, and microglial cluster formation in the brains of Siglec-11 tg mice at the 6- and 24-month age groups. Accordingly, we co-stained the hippocampus (Figure 1A) and substantia nigra pars reticulata (SN*pr*) (Figure 1C) with antibodies against Iba1 and CD68. In the hippocampus, the density of microglia, as determined by the number of Iba1-positive cells per selected area, was lower at 24 months of age in the Siglec-11 tg mice compared to WT control mice (Figure 1B). In detail, Siglec-11 tg mice showed substantially lower microglial density (84.05 ± 5.9%) at 24 months compared to WT mice (204.74 ± 12.7%; *p* < 0.001). We also analyzed Iba1 density in juvenile mice (1.5 months) to investigate whether the observed changes are possibly related to developmental abnormalities. However, there was no difference observed between WT and Siglec-11 tg mice in microglial density at 1.5 months of age (Supplementary Figure S2A). In addition, we studied the SN*pr* and found that the density of Iba1-positive cells was lower in the Siglec-11 tg mice compared to WT mice at 24 months of age (157.16 ± 31.5% vs. 227.19 ± 37%, *p* = 0.006) (Figure 1D). Again, analysis of 1.5-month-old mice showed no difference in the SN*pr* between the WT and Siglec-11 tg mice in microglial density (Supplementary Figure S2B).

Next, we analyzed the intensity of CD68 in the soma of microglia at different ages. Here, we found a lower microglial expression of CD68 in the hippocampus from 254 ± 44.2% in WT mice to 132.6 ± 20.8% in Siglec-11 tg mice (*p* = 0.009) at 24 months of age (Figure 1B), while no difference was observed in the substantia nigra (Figure 1D) at the same age.

**Figure 2:**
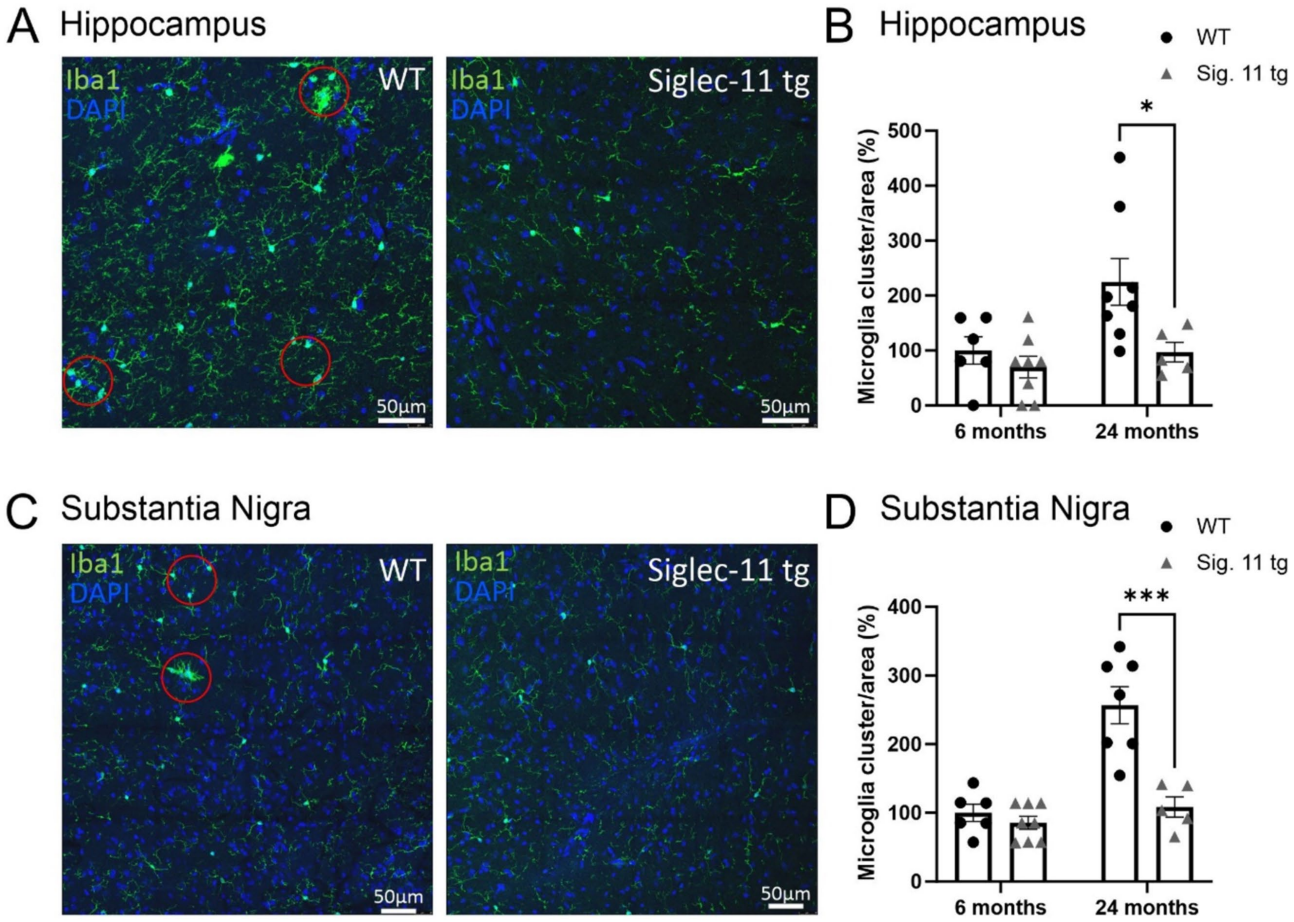
Less microglial cell clusters in aged Siglec-11 transgenic compared to wildtype mice. Hippocampal and substantia nigra tissue was stained with the antibody directed against the microglial transmembrane protein Iba1. Red circles measuring 50 μm in diameter elucidating the clustering of two or more microglial cells. **(A)** Representative images of Iba1 (green) and DAPI (blue) in the hippocampus of 24-month-old wildtype (WT) control and Siglec-11 transgenic (tg) mice are shown. **(B)** Quantification of hippocampal Iba1 cell clusters showed decreased levels at 24 months of age in Siglec-11 tg mice compared to WT mice. **(C)** Representative images of Iba1 (green) and DAPI (blue) in the substantia nigra *pars reticulata* of 24-month-old WT and Siglec-11 tg mice are shown. **(D)** Quantification in the substantia nigra *pars reticulata* showed decreased Iba1 cell clusters in Siglec-11 tg at 24 months of age compared to WT mice. Data were analyzed with two-way ANOVA and Fisher’s LSD *post hoc* test and are shown as mean ± SEM. *n* = 5–8 mice per group. Scale bars 50 μm. *p ≤ 0.05, ***p ≤ 0.001.

Finally, we analyzed the density of microglial cell clusters, which we defined as three or more microglial cell bodies in close proximity (within a radius of 50 μm) to each other (Figures 2A–D). While no difference was seen between both groups at 6 months of age, the density of microglial clusters was lower in Siglec-11 tg mice compared to WT mice at 24 months of age, both, in the hippocampus and the SN*pr*. In detail, at 24 months of age, hippocampal microglial cluster density was 96.73 ± 18% in Siglec-11 tg mice compared to 224.69 ± 42.6% in WT mice (*p* = 0.010) (Figure 2B), while in the SN*pr*, microglial cluster density was 108.41 ± 14.6% in Siglec-11 tg mice compared to 256.84 ± 27% in WT mice (*p* < 0.001) (Figure 2D).

Altogether, our data show lower microglial cell density and fewer microglial clusters in the hippocampus and SN*pr* at 24 months of age, and lower hippocampal lysosomal expression of CD68 in microglia at 24 months of age in Siglec-11 tg mice compared to WT mice.

### Less microglial lipid accumulation in the substantia nigra of Siglec-11 tg mice

**Figure 3:**
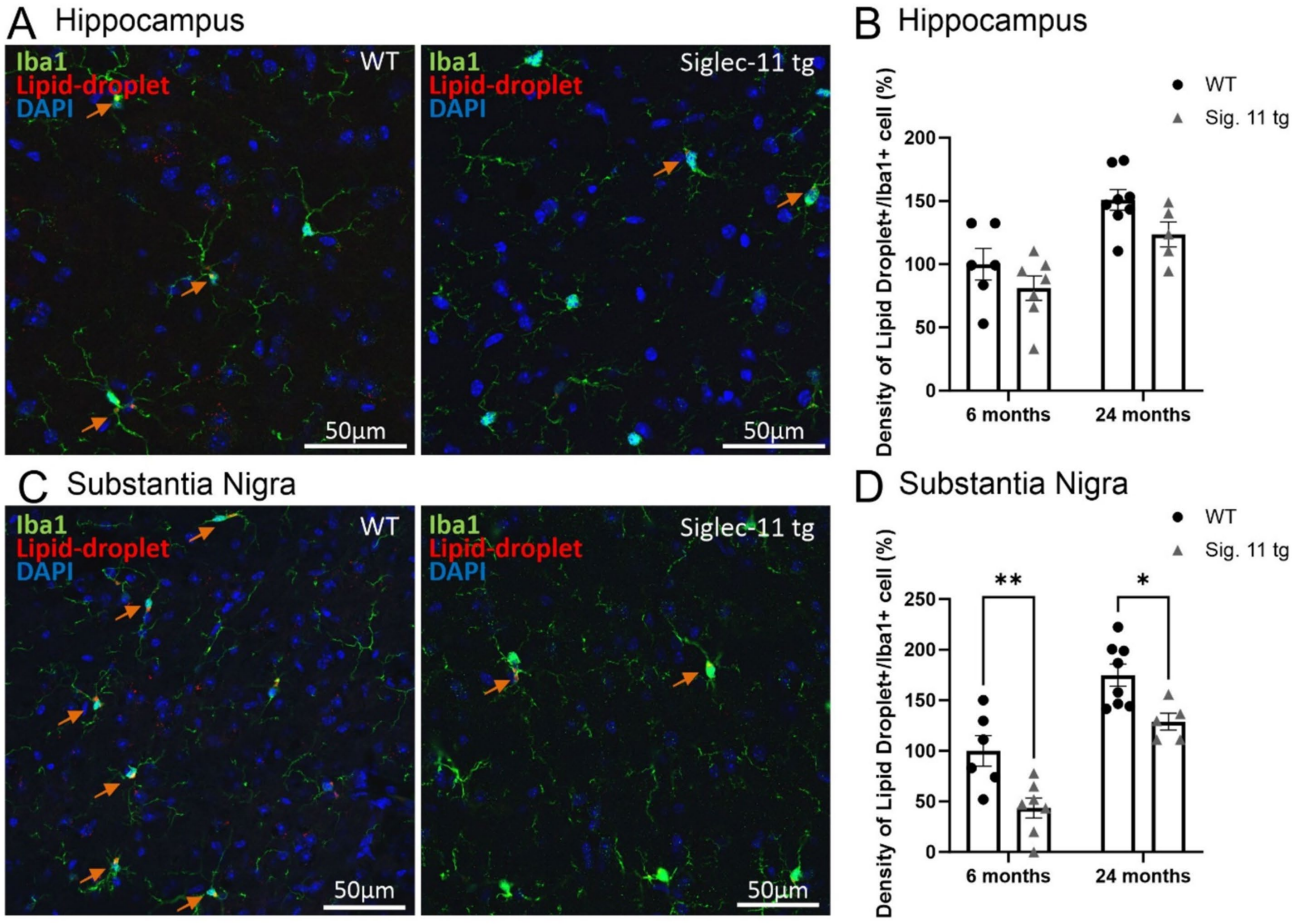
Decreased density of lipid-droplets containing microglia in the substantia nigra of Siglec-11 transgenic compared to wildtype mice. Hippocampal and substantia nigra tissue was stained with the antibody directed against the microglial transmembrane protein Iba1. **(A)** Representative images of lipid-droplets (red), Iba1 (green) and DAPI (blue) staining in the hippocampus at 24 months of age of wildtype (WT) and Siglec-11 transgenic (tg) mice. **(B)** Quantification of the number of lipid-droplets and Iba1 double-positive cells per area showed no significant change between Siglec-11 tg and WT control mice in the hippocampus. **(C)** Representative images of lipid-droplets (red), Iba1 (green), and DAPI (blue) staining in the substantia nigra *pars reticulata* (SN*pr*) at 24 months of age of WT and Siglec-11 tg mice. **(D)** Quantification of the number of lipid-droplets and Iba1 double-positive cells per area were decreased in the Siglec-11 tg mice at 6 and 24 months of age compared to WT control mice. Arrows pointing to microglial cells co-expressing lipid-droplet autofluorescence. Data were analyzed with two-way ANOVA and Fisher’s LSD *post hoc* test and are shown as mean ± SEM; *n* = 5–8 mice per group. Scale bars 50 μm. *p ≤ 0.05, ** *p* ≤ 0.01.

In addition, we investigated lipid accumulation in microglia as a hallmark of aging and oxidative stress in the brain (Fan et al., 2024). Therefore, we assessed the autofluorescence of lipid droplets in Iba1+ microglia in the hippocampus (Figure 3A) as well as in the SN*pr* (Figure 3C). Our data showed a general decrease in the density of lipid-laden (lipid droplets plus Iba1-positive cells) microglia in the SN*pr* of both 6- and 24-month-old Siglec-11 tg compared to WT mice, while no difference was seen in the hippocampus (Figures 3B,D). In detail, at 6 months of age, relative density of lipid-laden microglia in the SN*pr* differed from 100 ± 15% in WT mice to 43.55 ± 9.9% (*p* = 0.002) in Siglec-11 tg mice, and at 24 months of age from 174.80 ± 11% in WT mice to 128.83 ± 8.4% in Siglec-11 tg mice (*p* = 0.012; Figure 3D).

In summary, our data showed lower density of lipid-laden microglia in the SN*pr* of 6- and 24-month old Siglec-11 tg compared to WT mice.

### Higher neuronal density in the substantia nigra of aged Siglec-11 tg mice

**Figure 4:**
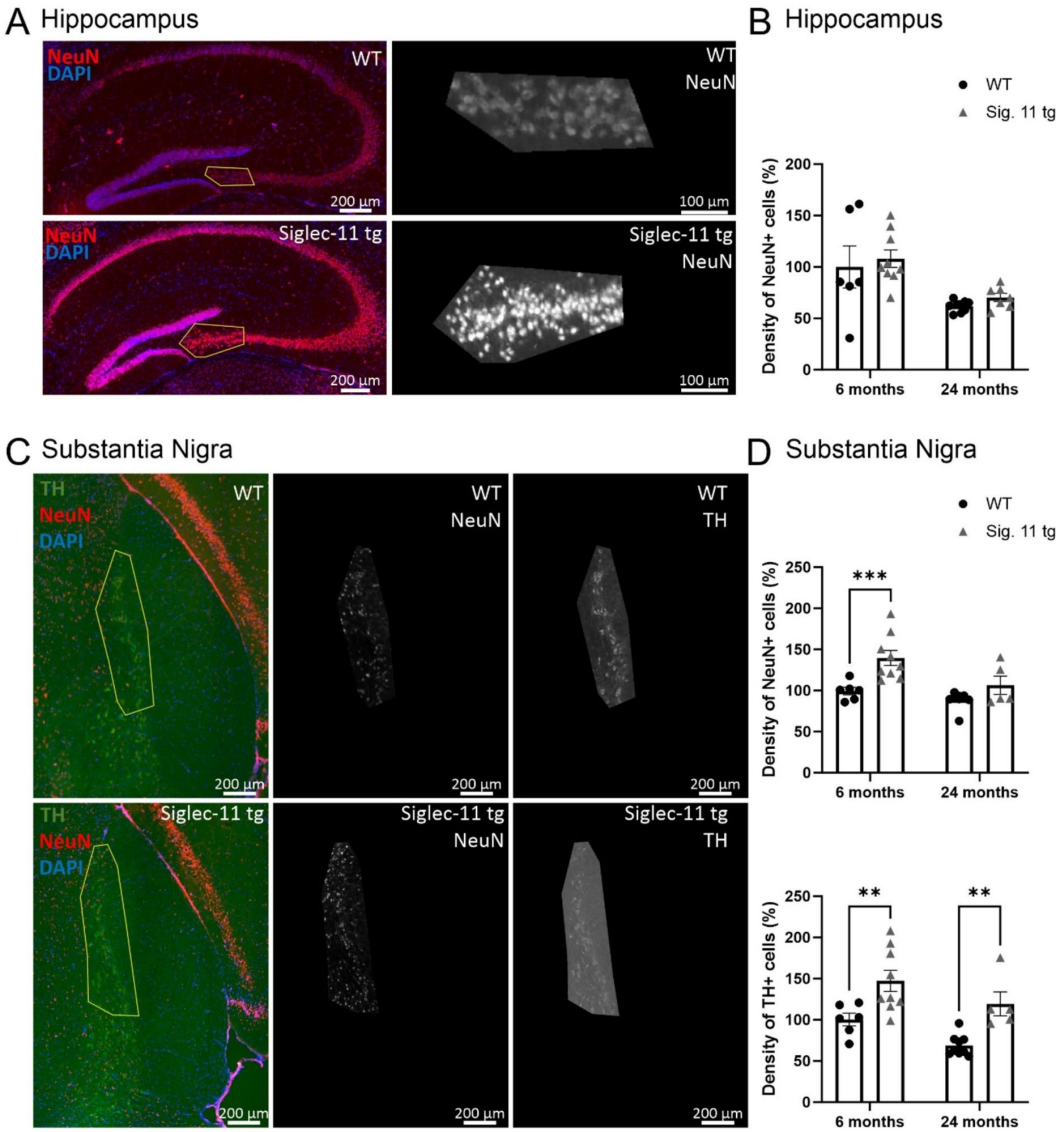
Higher number of dopaminergic neurons in the substantial nigra *pars compacta* in Siglec-11 transgenic compared to wildtype mice. Hippocampal tissue stained with the antibody directed against the neuronal nuclei (NeuN) and substantia nigra tissue stained with the antibodies directed against NeuN and the dopaminergic marker tyrosine hydroxylase (TH). **(A)** Representative images of NeuN (red) and DAPI (blue) staining in the hippocampus CA3 region of wildtype (WT) and Siglec-11 transgenic (tg) mice. **(B)** Quantification of NeuN-positive cell density in the hippocampal region showed no significant difference between WT and Siglec-11 tg mice. **(C)** Representative images of TH (green), NeuN (red), and DAPI (blue) staining in the substantia nigra *pars compacta* of 24-month-old WT and Siglec-11 tg mice. **(D)** Quantification of NeuN-positive cell density in the substantia nigra showed increased NeuN-positive cell density in Siglec-11 tg compared to WT mice at 6 months of age. Quantification of TH-positive cell density in the substantia nigra showed increased TH-positive cell density in Siglec-11 tg compared to WT mice at 6 and 24 months of age. Data were analyzed with two-way ANOVA and Fisher’s LSD *post hoc* test and are shown as mean ± SEM; *n* = 5–9 mice per group. Scale bars as indicated in the figures. **p ≤ 0.01, ***p ≤ 0.001.

To assess whether the decreased microglial activation observed in Siglec-11 tg mice has an impact on the neuronal numbers in the brain, neuronal density was quantified in the hippocampus and in the substantia nigra *pars compacta* (SN*pc*). Accordingly, we stained the hippocampal neurons with the neuronal nuclei marker (NeuN; Figure 4A), and in the SN*pc,* we used markers for NeuN and tyrosine hydroxylase (TH), a marker of dopaminergic neurons (Figure 4C). At 24 months of age, WT mice tended to have fewer numbers of hippocampal neurons in comparison to Siglec-11 tg mice, although it did not reach statistical significance (*p* = 0.531, Figure 4B). In the SN*pc*, we observed fewer NeuN-positive and TH-positive cells in WT mice compared to the Siglec-11 tg mice starting at 6 months of age, which became more prominently different for the TH-positive neurons at 24 months of age (Figure 4D). In detail, the relative NeuN-positive cells in the SN*pc* were lower in WT mice (100 ± 4.6%) compared to Siglec-11 tg mice (140 ± 9.2%, *p* < 0.001) at 6 months of age. Relative TH-positive cells were also fewer in WT (100 ± 7.7%) compared to Siglec-11 tg mice (147.3 ± 12.8%, *p* = 0.003) at 6 months of age, and between WT (68 ± 4%) and Siglec-11 tg mice (119 ± 14%, *p* = 0.003) at 24 months of age. We further quantified NeuN-positive cells in the hippocampus and NeuN-positive and TH-positive cells in the SN*pc* of 1.5-month-old juvenile mice to examine if the difference in neuronal numbers between both genotypes might already occur at an earlier time point. However, we found no significant differences in neuronal numbers at 1.5 months of age between WT and Siglec-11 tg mice (Supplementary Figures S3A,B).

Taken together, our results show more neuronal numbers in the SN*pc* of Siglec-11 tg compared to WT mice at 6 months of age, and prominent dopaminergic neuronal preservation in Siglec-11 tg mice at 24 months of age.

### Reduced oxidative phosphorylation and inflammatory gene transcript pathways in Siglec-11 tg mice

**Figure 5:**
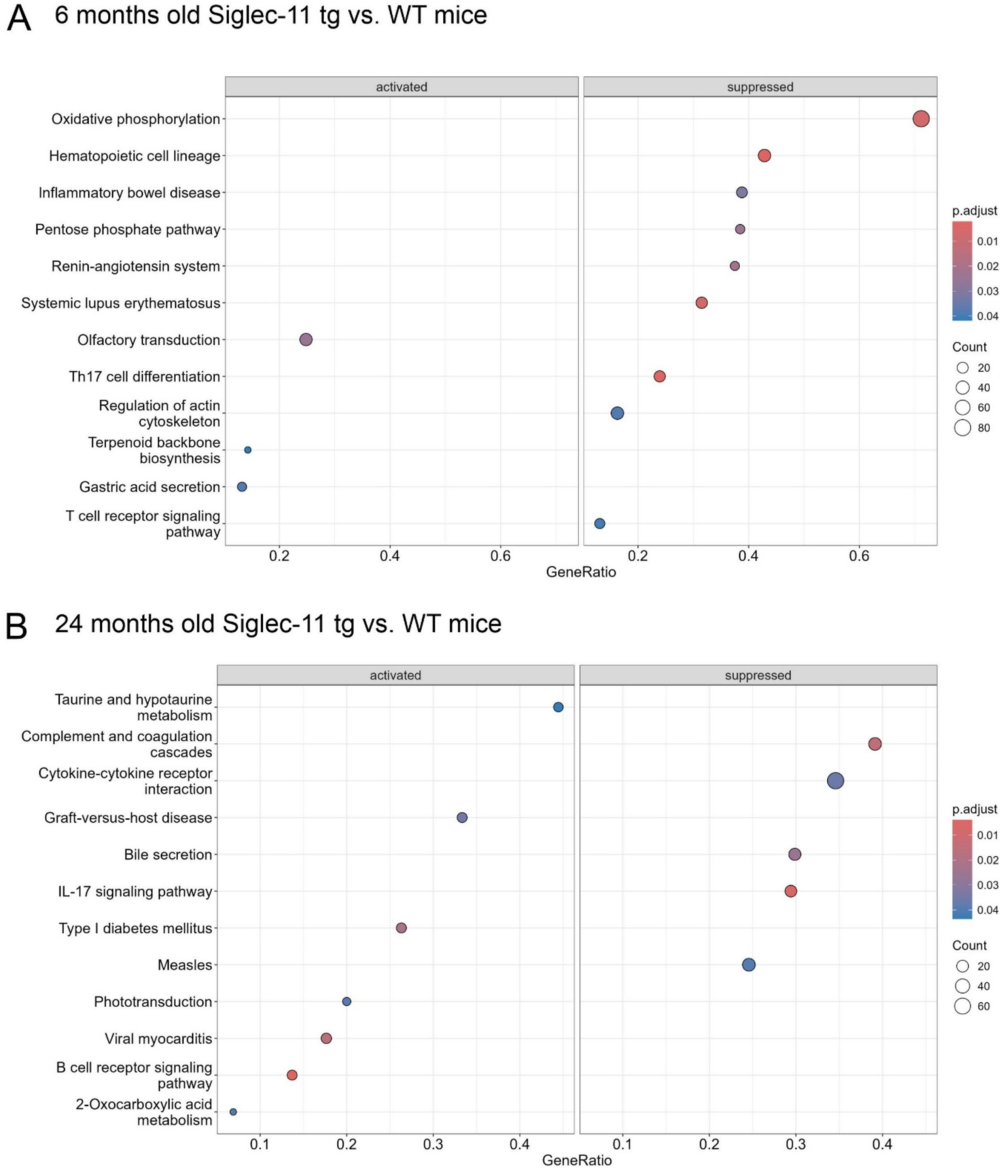
Suppressed oxidative phosphorylation, inflammatory, complement and coagulation transcriptome pathways in Siglec-11 transgenic compared to wildtype mice. Whole genome RNA sequencing of brain tissue was performed. Gene set enrichment on Kyoto encyclopedia of genes and genomes transcriptome pathway analysis was carried out with 6- and 24-month-old Siglec-11 transgenic (tg) in comparison to wildtype (WT) control mice of the same age. **(A)** At 6 months of age suppression of the oxidative phosphorylation and inflammatory pathways were observed in the Siglec-11 tg mice. **(B)** At 24 months of age suppression of complement and coagulation cascades, along with suppressed cytokine-cytokine receptor interaction pathways were found in the Siglec-11 tg mice. Data shown from *n* = 4–5 mice per group.

To better understand the underlying pathways related to the SIGLEC-11 receptor in the brain, and to unravel the mechanisms behind the observed changes in the Siglec-11 tg mice, we isolated RNA from the brain of 6- and 24-month-old Siglec-11 tg and WT mice and performed RNA-seq analysis. Principal component (PC) analysis showed a clear separation between the groups at both ages (Supplementary Figure S4A). For 6-month-old mice, PC1 accounted for 52% of the variance and PC2 accounted for 18% of the variance. We then performed a differentially expressed gene (DEG) analysis (Supplementary Figure S4B), which showed an increased expression of hemoglobin alpha and beta adult chain-related genes in the Siglec-11 tg mice compared to WT mice. On the other hand, mitochondrial ribosomal protein-related genes (*Mrps11* and *Mrpl46*), immune and inflammatory-related genes like S100 calcium-binding protein A9 (*S100a9*), cellular communication network factor 1 (*Ccn1*), ADAM metallopeptidase with thrombospondin type 1 motif 1 (*Adamts1*), and inhibitory synapses regulator gene neuronal PAS domain protein 4 (*Npas4*) were downregulated. We then performed a gene set enrichment analysis (GSEA) on Kyoto encyclopedia of genes and genomes (KEGG), which among hematopoietic cell lineage and inflammatory pathways, revealed the oxidative phosphorylation pathway to be the top suppressed pathway in the Siglec-11 tg mice compared to WT mice (Figure 5A). GSEA of mouse hallmark gene sets showed enrichment of 10 hallmark gene sets at FDR less than 25% and a nominal *p*-value of less than 0.05 in the WT mice compared to Siglec-11 tg mice. Out of these, the top enriched hallmark gene sets were the oxidative phosphorylation (nominal *p* < 0.02), along with the reactive oxygen species pathway (nominal *p* < 0.04), and apoptosis pathway (nominal *p* < 0.001; Supplementary Figure S4C).

The 24-month-old mice showed 38% variance on PC1 and 19% variance on PC2 in the PC analysis (Supplementary Figure S5A). The DEG analysis revealed *Snca* coding the alpha-synuclein protein (*α*-synuclein) as the top downregulated gene in Siglec-11 tg mice compared to WT mice (Supplementary Figure S5B). As seen in the 6-month group, the *Mrpl46* and *Mrps11* genes were also downregulated at 24 months in the Siglec-11 tg mice. The Homeobox B8 (*Hoxb8*) gene was the only significantly upregulated gene in Siglec-11 tg mice at 24 months of age. Gene set enrichment analysis (GSEA) on KEGG showed the suppression of complement and coagulation cascades along with cytokine-cytokine receptor interaction in Siglec-11 tg mice compared to WT mice (Figure 5B). GSEA of hallmark gene sets showed three enriched gene sets at a nominal *p*-value of less than 0.05 in WT mice compared to Siglec-11 tg mice, which were angiogenesis, Kirsten rat sarcoma virus (KRAS) signaling, and epithelial-mesenchymal transition. Complement and inflammatory gene sets were also enriched in WT compared to Siglec-11 tg mice, although not reaching the significance threshold (Supplementary Figure S5C).

Taken together, the RNA-seq analysis showed suppressed oxidative stress and apoptotic pathways at 6 months of age and suppressed complement and inflammatory pathways at 24 months of age in Siglec-11 tg compared to WT mice.

### Decreased gene transcription of microglial and astrocytic markers in aged Siglec-11 tg mice

**Figure 6:**
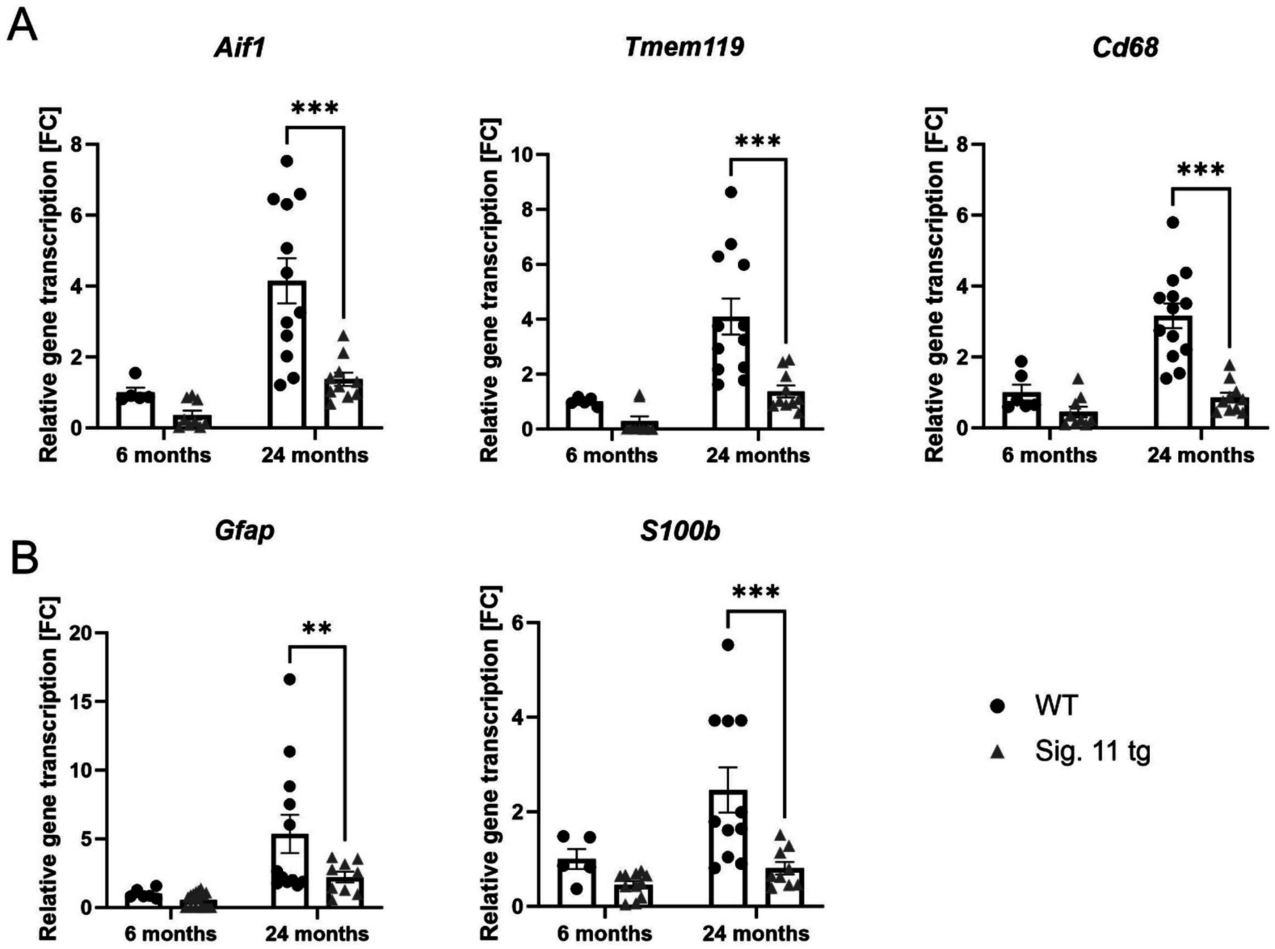
Decreased gene transcription of microglial and astrocytic markers in aged Siglec-11 transgenic compared to WT mice. Semi-quantitative real-time PCR (sqRT-PCR) of microglial and astrocytic markers in brain hemisphere homogenates of Siglec-11 transgenic (tg) and wildtype (WT) control mice. **(A)** Transcription of the microglial markers *Aif1* (allograft inflammatory factor 1) and *Tmem119* (transmembrane protein 119), along with the microglial activation marker *Cd68* showed decreased levels at 24-months of age in Siglec-11 tg mice compared to WT control mice. **(B)** Transcription of the astrocytic markers *Gfap* (glial fibrillary acidic protein) and *S100b* (S100 calcium-binding protein B) showed decreased levels of *S100b* at 24-months of age in Siglec-11 tg mice compared to WT control mice. Data were analyzed with two-way ANOVA and Fisher’s LSD *post hoc* test and are shown as mean ± SEM. *n* = 5–18 mice per group. **p ≤ 0.01, ***p ≤ 0.001.

Next, we measured the gene transcription levels of the macrophage/microglial markers allograft inflammatory factor 1 (*Aif1*) and transmembrane protein 119 (*Tmem119*), along with the macrophage lysosomal activation marker *Cd68,* in the whole-brain hemisphere homogenates of Siglec-11 tg mice and WT controls (Figure 6A). Siglec-11 tg mice showed lower gene transcription levels of all microglial markers in comparison to WT mice. In detail, at 24 months of age, the relative *Aif1* gene transcription differed from 4.15 ± 0.6 fold change (FC) in WT mice to 1.37 ± 0.2 FC (*p* < 0.001) in Siglec-11 tg mice, *Tmem119* differed from 4.10 ± 0.7 FC to 1.37 ± 0.2 FC (*p* < 0.001), and *Cd68* differed from 3.16 ± 0.3 FC to 0.86 ± 0.1 FC (*p* < 0.001), respectively.

We also tested the astrocytic markers glial fibrillary acidic protein (*Gfap*) and S100 calcium-binding protein B (*S100b*; Figure 6B). In comparison to WT at 24 months of age, Siglec-11 tg mice showed a lower *Gfap* gene transcription, differing from 5.36 ± 1.4 FC in WT mice to 2.21 ± 0.4 FC in Siglec-11 tg mice (*p* = 0.009), and *S100b* gene transcription, differed from 2.46 ± 0.5 FC in WT mice to 0.80 ± 0.1 FC in Siglec-11 tg mice (*p* < 0.001). We tested the microglial and astrocytic markers at the younger age of 1.5 months and found no difference between both genotype groups (Supplementary Figures S6A,B).

In summary, our data showed decreased gene transcription of microglial and astrocytic markers in 24-month-old Siglec-11 tg compared to WT mice.

### Decreased inflammatory, oxidative burst, and complement gene transcripts in aged Siglec-11 tg mice

**Figure 7:**
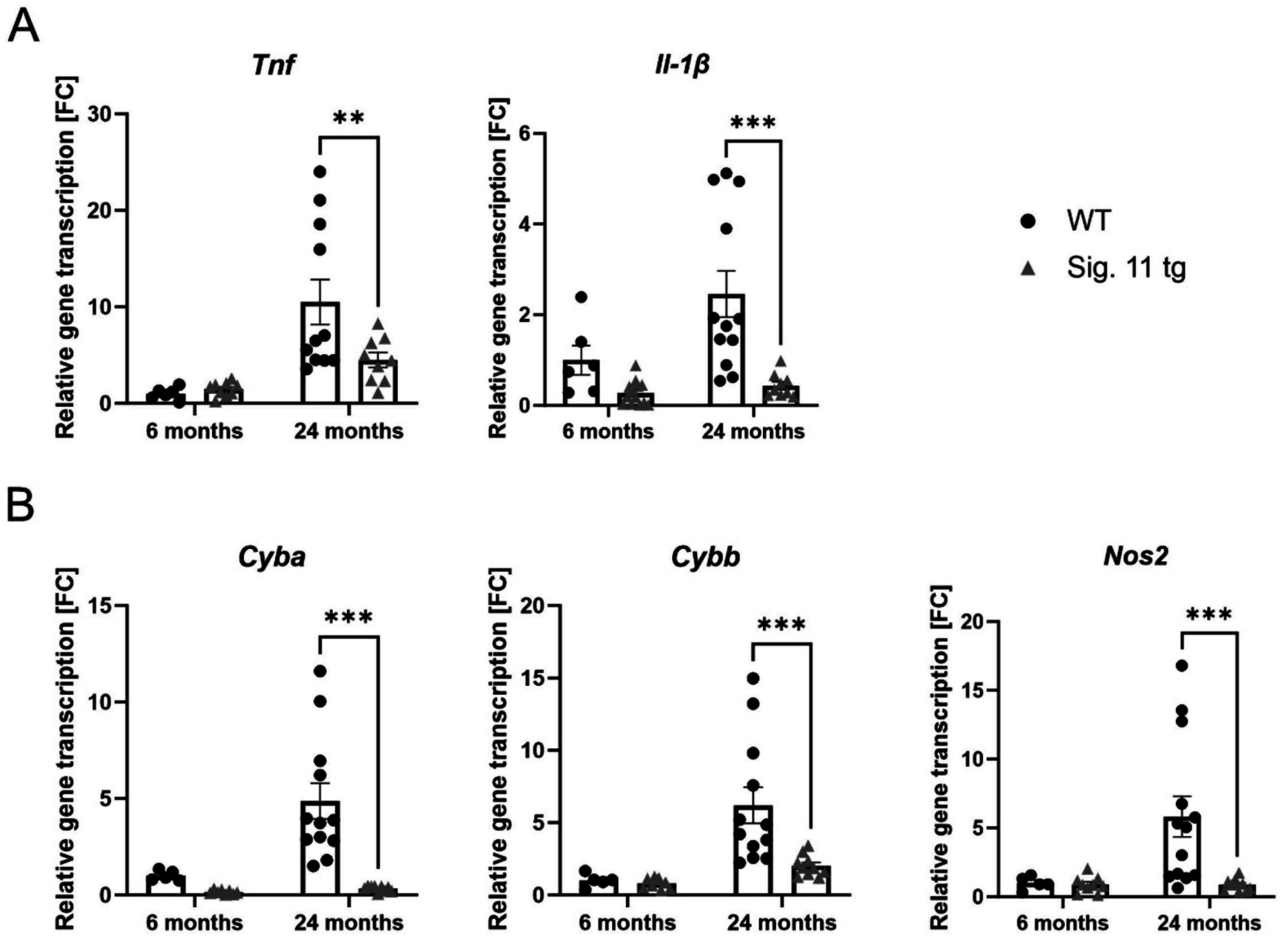
Decreased transcription of pro-inflammatory cytokines and oxidative stress markers in aged Siglec-11 transgenic compared to wildtype mice. Semi-quantitative real-time PCR (sqRT-PCR) of brain hemisphere homogenates of Siglec-11 transgenic (tg) and wildtype (WT) control mice. **(A)** Transcription of the pro-inflammatory cytokines *tumor necrosis factor-α* (*Tnf*) and *interleukin-1β* (*Il-1β*) were decreased in Siglec-11 tg mice in comparison to WT control mice at 24-months of age. **(B)** Transcription levels of *cytochrome b-245 alpha and beta* (*Cyba* and *Cybb*) and the *nitric oxide synthase 2* (*Nos2*) were decreased in 24-month-old Siglec-11 tg mice compared to WT mice. Data were analyzed with two-way ANOVA and Fisher’s LSD *post hoc* test and are shown as mean ± SEM. *n* = 5–13 mice per group. **p ≤ 0.01, ***p ≤ 0.001.

**Figure 8:**
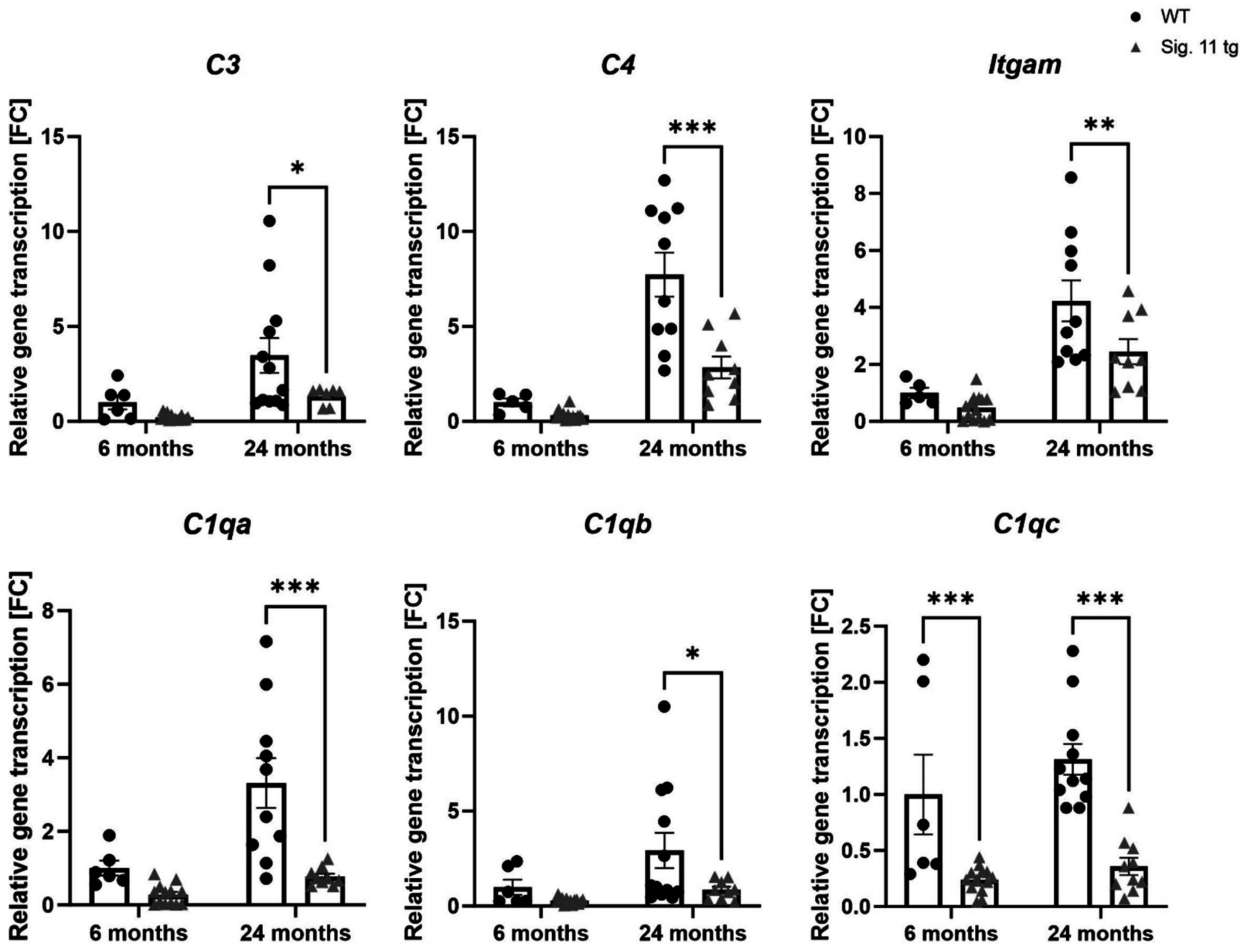
Decreased transcription of complement system associated factors in aged Siglec-11 transgenic compared to wildtype mice. Semi-quantitative real-time PCR (sqRT-PCR) of brain hemisphere homogenates of Siglec-11 transgenic (tg) and wildtype (WT) control mice. Transcription levels of complement components 3 and 4 (*C3* and *C4*), Integrin alpha M (*Itgam*), and complement C1q subcomponent subunits A and C (*C1qa* and *C1qc*) were decreased in 24-month-old Siglec-11 tg mice compared to WT mice. *C1qc* was also decreased in 6-month-old Siglec-11 tg mice compared to WT mice. Data were analyzed with two-way ANOVA and Fisher’s LSD *post hoc* test and are shown as mean ± SEM. *n* = 5–14 mice per group. *p ≤ 0.05, **p ≤ 0.01, ***p ≤ 0.001.

To affirm the results obtained from the RNA-seq analysis and to examine whether the reduced microglial activation in Siglec-11 tg mice also leads to reduced inflammatory/immune activated states in comparison to WT mice, we measured the gene expression levels of different pro-inflammatory, oxidative burst, and complement system markers via sqRT-PCR (Figures 7, 8). We investigated the transcription levels of the pro-inflammatory cytokines tumor necrosis factor alpha (*Tnf*) and interleukin-1ß (*Il-1ß*) (Figure 7A). In comparison to WT, Siglec-11 tg mice showed a lower gene transcription of *Tnf*, differing from 10.51 ± 2.3 FC in WT mice to 4.48 ± 0.8 FC in Siglec-11 tg mice (*p* = 0.006), and lower *Il-1ß* gene transcription, differing from 2.46 ± 0.5 FC in WT mice to 0.44 ± 0.1 FC in Siglec-11 tg mice (*p* < 0.001) at 24 months of age. We tested both markers at 1.5 months of age and found no difference between the groups (Supplementary Figure S6C).

To explore the effect of the SIGLEC-11 receptor on oxidative damage in the brain, we measured the gene transcript levels of the oxidative stress markers cytochrome b-245 alpha and beta (*Cyba* and *Cybb*) and the nitric oxide synthase 2 (*Nos2*) by sqRT-PCR (Figure 7B). Our results showed clearly lower gene transcription of these oxidative stress markers in the Siglec-11 tg mice compared to WT mice. In detail, at 24 months of age *Cyba*, *Cybb,* and *Nos2* were all higher in WT mice compared to Siglec-11 tg mice, differing from 4.87 ± 0.9 FC to 0.33 ± 0.1 FC (*p* < 0.001), from 6.2 ± 1.2 FC to 2.03 ± 0.2 FC (*p* < 0.001), and from 5.83 ± 1.5 FC to 0.87 ± 0.1 FC (*p* < 0.001), respectively. Analyzing all three markers at 1.5 months of age revealed a higher *Cyba* gene transcription in WT mice compared to Siglec-11 tg mice, differing from 0.44 ± 0.02 FC to 0.02 (*p* < 0.001), while both *Cybb* and *Nos2* were lower in WT mice compared to Siglec-11 tg mice, differing from 0.45 ± 0.03 FC to 0.98 ± 0.1 FC (*p* < 0.001) and from 1.7 ± 0.1 FC to 2.3 ± 0.1 FC (*p* = 0.001), respectively (Supplementary Figure S6D).

We then analyzed the gene transcript levels of complement components 3 and 4 (*C3* and *C4*) along with the integrin alpha M (*Itgam*) and the complement C1q subcomponent subunits a-c (*C1qa*, *C1qb*, *C1qc*) to unravel the effect of SIGLEC-11 receptor on the complement system activation (Figure 8). We observed a general decrease of complement system genes in Siglec-11 tg mice compared to WT mice. In detail, at 24 months of age *C3* differed from 3.48 ± 0.9 FC in WT mice to 1.33 ± 0.2 FC (*p* < 0.018) in Siglec-11 tg mice, *C4* differed from 7.73 ± 1.2 FC in WT mice to 2.85 ± 0.6 FC (*p* < 0.001) in Siglec-11 tg mice, and *Itgam* differed from 4.23 ± 0.7 FC in WT mice to 2.44 ± 0.4 FC in Siglec-11 tg mice (*p* = 0.007). The gene transcript levels of complement C1q subcomponents were lower in Siglec-11 transgenic mice compared to WT mice at 24 months of age, differing from 0.77 ± 0.1 FC to 3.31 ± 0.7 FC (*p* < 0.001) for *C1qa*, from 0.85 ± 0.2 FC to 2.93 ± 0.9 FC (*p* = 0.014) for *C1qb,* and from 0.36 ± 0.1 FC to 1.31 ± 0.1 FC (*p* < 0.001), respectively. *C1qc* was also lower in Siglec-11 tg mice compared to WT mice at 6 months of age, differing from 1.00 ± 0.4 FC to 0.24 ± 0.03 FC (*p* < 0.001). Analysis of complement markers gene transcripts at 1.5 months of age revealed a lower *C4* and a higher *Itgam* transcript level in WT mice compared to Siglec-11 tg mice, differing from 0.06 ± 0.01 FC to 0.25 ± 0.04 FC (*p* < 0.001), and from 2.29 ± 0.1 FC to 1.79 ± 0.08 FC (*p* = 0.002), respectively (Supplementary Figure S6E).

Overall, our results show a lower gene expression of inflammatory, oxidative stress, and complement system markers in 24-month-old Siglec-11 tg mice compared to WT mice, which also exhibited lower *C1qc* gene transcription at 6 months of age.

## Discussion

Sialic acid-binding immunoglobulin-type lectin (SIGLEC) receptors of microglial cells and tissue macrophages recognize intact sialylation, acting as innate immune checkpoints to regulate inflammation and oxidative stress in the central nervous system (Klaus et al., 2021; Liao et al., 2020).

Recent studies, have identified SIGLEC-11 as the most significant microglial gene associated with Alzheimer’s disease (AD), following the categorization of AD and dementia-related genes based on their relevance to the disease process (Bellenguez et al., 2022). Genomic data suggest that in humans, both *SIGLEC11* and the closely related *SIGLEC16* gene likely originated through gene conversion from the non-functional pseudogene *SIGLEC16P* (Hayakawa et al., 2017; Wang et al., 2012). This event is believed to have led to the exclusive expression of SIGLEC-11 in human microglia. Notably, microglial SIGLEC-11 in the human brain appears to exist solely as an alternative splice variant, which lacks the exon encoding the final (fifth) Ig-like C2-set domain within the protein’s extracellular segment, a characteristic shared by the humanized Siglec-11 transgenic mice used in this study (Hane et al., 2021; Wang and Neumann, 2010).

Activation of microglial SIGLEC-11 by polysialic acid (polySia) attached to neural cell adhesion molecule (NCAM) on neighboring neurons suppressed lipopolysaccharide (LPS)-induced pro-inflammatory mediators, such as *Il-1ß,* in a murine neuron–microglia co-culture, demonstrating the neuroprotective effect of SIGLEC-11 (Wang and Neumann, 2010). Soluble polySia has also been explored therapeutically, targeting the SIGLEC-11 receptor in humanized Siglec-11 transgenic mice (Karlstetter et al., 2017; Liao et al., 2021). In an age-related macular degeneration animal model, intravitreal application of soluble polySia in humanized Siglec-11 transgenic mice reduced mononuclear phagocytes and vascular leakage, and prevented complement system activation (Karlstetter et al., 2017). In a Parkinson’s disease model using the same humanized Siglec-11 tg mice, intraperitoneal administration of soluble polySia reduced microglial immunoreactivity and protected against dopaminergic neuronal degeneration in the substantia nigra (Liao et al., 2021).

This study focused on the role of SIGLEC-11 receptor in normal brain aging, comparing aged 24-month-old humanized Siglec-11 tg mice to wild-type (WT) mice. As a baseline, we examined mature 6-month-old adult mice. Additionally, we analyzed 1.5-month-old juvenile mice to investigate potential developmental effects but found no significant developmental differences between Siglec-11 tg and WT mice at that age.

Under steady-state conditions, microglia typically exhibit a quiescent immune profile and only become activated when stimulated, but increased microglial activation has been observed in aging brains across various species (Luo et al., 2010). Microglial density has also been shown to increase with age in CNS regions such as the hippocampus, visual and auditory cortices, and the retina (Damani et al., 2011; Mouton et al., 2002; Tremblay et al., 2012). Additionally, increased microglial clustering is seen in aging brains, especially near senile plaques in humans (Dickson et al., 1992) and in the brains of 24-month-old mice (Raj et al., 2017). Microglial activation is further evidenced by increased expression of the lysosomal CD68 microglial activation marker in aging mice brains (Halder and Milner, 2022).

In line with these observations, our study revealed a reduction in Iba1-positive microglial cell density and clustering in the hippocampus and substantia nigra of 24-month-old Siglec-11 tg compared to age-matched WT controls. We also noted reduced microglial phagolysosomal activation in the hippocampus of Siglec-11 tg mice compared to WT control mice at 24 months of age, as depicted by lower CD68 intensity per soma.

Dopaminergic neuronal loss in the substantia nigra *pars compacta* (SN*pc*) of aging C57BL/6 mice has been previously reported (Noda et al., 2020). Consistently, we observed a reduction in dopaminergic neuron density in aged 24-month-old WT control mice, whereas dopaminergic neurons were preserved in the SN*pc* of the Siglec-11 tg mice. This suggests that microglial SIGLEC-11 receptor protects against age-related neuronal loss in the substantia nigra.

The normal aging process is associated with numerous subtle and selective changes in the brain, often linked to cognitive decline (Burke and Barnes, 2006). Prominent changes include alterations in synaptic physiology, mitochondrial metabolism, and oxidative phosphorylation (Bishop et al., 2010; Castelli et al., 2019; Yankner et al., 2008). Our RNA sequencing and transcriptomic KEGG pathway analyses revealed oxidative phosphorylation as the most suppressed gene transcript pathway in 6-month-old Siglec-11 tg mice compared to WT controls. In contrast, pathways associated with apoptosis, inflammation, coagulation, and oxidative stress were enriched in WT mice compared to Siglec-11 tg mice at 6 months of age. By the age of 24 months, KEGG pathway analysis of Siglec-11 tg mice showed suppression of coagulation, complement cascade, and cytokine-cytokine receptor interaction pathways compared to WT mice. Here, the discrepancy between the enriched pathways at different age groups could be related to the whole genome transcriptome approach or the heterogeneous nature of the brain tissue, which was collected at different time points. The current study also suggests to use in future a cell-specific single-cell transcriptomic analysis of microglia isolated from different age groups at the same time point that should allow to much better illustrate the effect of age on microglial-specific pathways. Similar transcriptome studies also demonstrated that brain aging in mice is associated with an inflammatory response, oxidative stress, and synaptic alterations (Jiang et al., 2001; Lee et al., 2000). Our data also showed that *Snca*, which encodes the alpha-synuclein (*α*-synuclein) protein, was the top downregulated gene in 24-month-old Siglec-11 tg mice. Increased α-synuclein expression and its accumulation in the aging brain have been implicated in cognitive and motor dysfunction, particularly in Parkinson’s disease (Xuan et al., 2011; Yang et al., 2020).

Previous studies have reported increased expression of *Cd68* and elevated levels of pro-inflammatory cytokines, such as *Tnf*, *Il-1ß*, *Il-6*, and *Il-10,* in aged microglia (Griffin et al., 2006; Wong et al., 2005; Antignano et al., 2023). Specifically, the Trem2/Dap12 signaling pathway has been identified as critical for inducing the activated, phagocytic microglial phenotype (Konishi and Kiyama, 2020). Moreover, a study examining the aging microglial signature demarcated the Fc Fragment of IgE receptor Ig (FCER1G) as a microglial biomarker associated with aging and neurodegeneration (Mukherjee et al., 2019). In aged 24-month-old Siglec-11 tg mice, we observed a lower inflammatory profile and reduced immune activation compared to WT controls, with decreased gene transcription of microglial markers *Aif1* and *Tmem119,* as well as inflammatory markers *Tnf* and *Il1ß,* as demonstrated by sqRT-PCR. Additionally, 24-month-old Siglec-11 tg mice exhibited lower gene transcription of the phagocytic marker *Cd68* compared to WT mice, reflecting a more youthful microglial transcript profile.

Oxidative stress is a hallmark of both cognitive aging and neurodegenerative diseases, as neurons are particularly vulnerable to oxidative damage (Ionescu-Tucker and Cotman, 2021). SIGLECs have been shown to prevent oxidative stress-related aging. For instance, Siglec-E knockout mice displayed a reduced maximum lifespan, signs of oxidative damage, and accelerated aging (Schwarz et al., 2015). In our study, we examined the effect of the human microglial SIGLEC-11 receptor on oxidative stress gene transcript markers in the aging brain. Our sqRT-PCR analysis demonstrated that brains of aged Siglec-11 tg mice had lower gene transcription of phagocytosis-associated oxidative burst markers like *Cyba*, *Cybb*, and *Nos2* in comparison to WT controls. This further validated our RNA sequencing findings of suppressed oxidative phosphorylation pathways shown in the Siglec-11 tg mice. Moreover, we were able to validate our RNA sequencing findings of suppressed complement system pathways in the aged Siglec-11 tg mice by showing decreased gene transcription of complement markers *C3*, *C4*, and *Itgam* by sqRT-PCR. It is important to note that the decrease in inflammatory, complement, and oxidative stress gene markers observed in Siglec-11 tg mice also could be a consequence of reduced microglial numbers. However, the observed reduction in inflammation, with decreased microglial numbers, is clearly associated with a protective phenotype of Siglec-11 tg mice with increased neuronal density in the substantia nigra.

Lipid accumulation and droplet formation in microglia have been implicated in brain aging and neurodegeneration (Haney et al., 2024; Shimabukuro et al., 2016). Lipid droplets have typical autofluorescence properties that can be easily monitored by characteristic wavelength of confocal laser scanning microscopy. We determined the density of lipid-laden microglia and showed that Siglec-11 tg mice had less lipid-laden microglia in the substantia nigra at 6- and 24-months of age compared to WT controls.

In summary, aged 24-month-old humanized Siglec-11 tg mice exhibited a less aged brain phenotype compared to WT controls. They lacked the typical age-related increase in microglial density, CD68 upregulation, and microglial clustering. Additionally, mature Siglec-11 tg mice showed decreased lipid-laden microglial accumulation, suppression of oxidative stress and inflammatory pathways at 6 months of age, and suppression of complement and coagulative pathways at 24 months of age compared to WT controls. Particularly, gene transcripts of the pro-inflammatory cytokines *Tnf* and *Il-1β*, complement components *C3*, *C4,* and *Itgam*, and oxidative stress markers *Cyba*, *Cybb*, and *Nos2* were decreased in aged Siglec-11 tg mice. In addition, aged Siglec-11 tg mice presented less age-related neuronal loss in the substantia nigra at 24 months compared to WT mice. Collectively, these findings suggest that microglial SIGLEC-11 receptors play a crucial role in protecting the brain from “inflammaging.”

## Data Availability

The datasets presented in this study can be found in online repositories. The names of the repository/repositories and accession number(s) can be found at: www.ncbi.nlm.nih.gov/geo, GSE282391.

## References

[ref1] Angata T. Kerr S. C. Greaves D. R. Varki N. M. Crocker P. R. Varki A. (2002). Cloning and characterization of human Siglec-11. A recently evolved signaling molecule that can interact with SHP-1 and SHP-2 and is expressed by tissue macrophages, including brain microglia. J. Biol. Chem. 277, 24466–24474. doi: 10.1074/JBC.M202833200, PMID: 11986327

[ref2] Antignano I. Liu Y. Offermann N. Capasso M. (2023). Aging microglia. Cell. Mol. Life Sci. 80:126. doi: 10.1007/S00018-023-04775-Y, PMID: 37081238 PMC10119228

[ref3] Bellenguez C. Küçükali F. Jansen I. E. Kleineidam L. Moreno-Grau S. Amin N.. (2022). New insights into the genetic etiology of Alzheimer’s disease and related dementias. Nat. Genet. 54, 412–436. doi: 10.1038/S41588-022-01024-Z, PMID: 35379992 PMC9005347

[ref4] Bishop N. A. Lu T. Yankner B. A. (2010). Neural mechanisms of ageing and cognitive decline. Nature 464, 529–535. doi: 10.1038/NATURE08983, PMID: 20336135 PMC2927852

[ref9001] Bodea L. G. Wang Y. Linnartz-Gerlach B. Kopatz J. Sinkkonen L. Musgrove R.. (2014). Neurodegeneration by Activation of the Microglial Complement–Phagosome Pathway. J Neurosci. 34:8546. doi: 10.1523/JNEUROSCI.5002-13.2014 24948809 PMC6608212

[ref5] Burke S. N. Barnes C. A. (2006). Neural plasticity in the ageing brain. Nat. Rev. Neurosci. 7, 30–40. doi: 10.1038/NRN1809 16371948

[ref6] Bushnell B. Rood J. Singer E. (2017). BBMerge – accurate paired shotgun read merging via overlap. PLoS One 12:e0185056. doi: 10.1371/JOURNAL.PONE.0185056, PMID: 29073143 PMC5657622

[ref7] Carlson M. (2019). Bioconductor – org.Mm.eg.db. Available at: 10.18129/B9.bioc.org.Mm.eg.db (Accessed July 8, 2024).

[ref8] Castelli V. Benedetti E. Antonosante A. Catanesi M. Pitari G. Ippoliti R.. (2019). Neuronal cells rearrangement during aging and neurodegenerative disease: metabolism, oxidative stress and organelles dynamic. Front. Mol. Neurosci. 12:132. doi: 10.3389/FNMOL.2019.00132, PMID: 31191244 PMC6546816

[ref9] Damani M. R. Zhao L. Fontainhas A. M. Amaral J. Fariss R. N. Wong W. T. (2011). Age-related alterations in the dynamic behavior of microglia. Aging Cell 10, 263–276. doi: 10.1111/J.1474-9726.2010.00660.X, PMID: 21108733 PMC3056927

[ref10] Dickson D. W. Crystal H. A. Mattiace L. A. Masur D. M. Blau A. D. Davies P.. (1992). Identification of normal and pathological aging in prospectively studied non-demented elderly humans. Neurobiol. Aging 13, 179–189. doi: 10.1016/0197-4580(92)90027-U, PMID: 1311804

[ref11] Dobin A. Davis C. A. Schlesinger F. Drenkow J. Zaleski C. Jha S.. (2013). STAR: ultrafast universal RNA-seq aligner. Bioinformatics 29, 15–21. doi: 10.1093/BIOINFORMATICS/BTS635, PMID: 23104886 PMC3530905

[ref12] Fan H. Zhang M. Wen J. Wang S. Yuan M. Sun H.. (2024). Microglia in brain aging: an overview of recent basic science and clinical research developments. J. Biomed. Res. 38, 122–136. doi: 10.7555/JBR.37.20220220, PMID: 38403286 PMC11001587

[ref13] Griffin R. Nally R. Nolan Y. McCartney Y. Linden J. Lynch M. A. (2006). The age-related attenuation in long-term potentiation is associated with microglial activation. J. Neurochem. 99, 1263–1272. doi: 10.1111/J.1471-4159.2006.04165.X, PMID: 16981890

[ref14] Halder S. K. Milner R. (2022). Exaggerated hypoxic vascular breakdown in aged brain due to reduced microglial vasculo-protection. Aging Cell 21:e13720. doi: 10.1111/ACEL.13720, PMID: 36130175 PMC9649604

[ref15] Hane M. Chen D. Y. Varki A. (2021). Human-specific microglial Siglec-11 transcript variant has the potential to affect polysialic acid-mediated brain functions at a distance. Glycobiology 31, 231–242. doi: 10.1093/GLYCOB/CWAA082, PMID: 32845322 PMC8022978

[ref16] Haney M. S. Pálovics R. Munson C. N. Long C. Johansson P. K. Yip O.. (2024). APOE4/4 is linked to damaging lipid droplets in Alzheimer’s disease microglia. Nature 628, 154–161. doi: 10.1038/s41586-024-07185-7, PMID: 38480892 PMC10990924

[ref17] Hayakawa T. Angata T. Lewis A. L. Mikkelsen T. S. Varki N. M. Varki A. (2005). A human-specific gene in microglia. Science 309, 754–755. doi: 10.1126/SCIENCE.1114321 16151003

[ref18] Hayakawa T. Khedri Z. Schwarz F. Landig C. Liang S. Y. Yu H.. (2017). Coevolution of Siglec-11 and Siglec-16 via gene conversion in primates. BMC Evol. Biol. 17:228. doi: 10.1186/S12862-017-1075-Z, PMID: 29169316 PMC5701461

[ref19] Howell R. D. Dominguez-Lopez S. Ocañas S. R. Freeman W. M. Beckstead M. J. (2020). Female mice are resilient to age-related decline of substantia nigra dopamine neuron firing parameters. Neurobiol. Aging 95, 195–204. doi: 10.1016/J.NEUROBIOLAGING.2020.07.025, PMID: 32846275 PMC7606778

[ref20] Ionescu-Tucker A. Cotman C. W. (2021). Emerging roles of oxidative stress in brain aging and Alzheimer’s disease. Neurobiol. Aging 107, 86–95. doi: 10.1016/J.NEUROBIOLAGING.2021.07.014 34416493

[ref21] Jiang C. H. Tsien J. Z. Schultz P. G. Hu Y. (2001). The effects of aging on gene expression in the hypothalamus and cortex of mice. Proc. Natl. Acad. Sci. USA 98, 1930–1934. doi: 10.1073/PNAS.98.4.1930, PMID: 11172053 PMC29359

[ref22] Karlstetter M. Kopatz J. Aslanidis A. Shahraz A. Caramoy A. Linnartz-Gerlach B.. (2017). Polysialic acid blocks mononuclear phagocyte reactivity, inhibits complement activation, and protects from vascular damage in the retina. EMBO Mol. Med. 9, 154–166. doi: 10.15252/emmm.201606627, PMID: 28003336 PMC5286381

[ref23] Khan N. Kim S. K. Gagneux P. Dugan L. L. Varki A. (2020). Maximum reproductive lifespan correlates with CD33rSIGLEC gene number: implications for NADPH oxidase-derived reactive oxygen species in aging. FASEB J. 34, 1928–1938. doi: 10.1096/FJ.201902116R, PMID: 31907986 PMC7018541

[ref24] Klaus C. Liao H. Allendorf D. H. Brown G. C. Neumann H. (2021). Sialylation acts as a checkpoint for innate immune responses in the central nervous system. Glia 69, 1619–1636. doi: 10.1002/GLIA.23945, PMID: 33340149

[ref25] Konishi H. Kiyama H. (2020). Non-pathological roles of microglial TREM2/DAP12: TREM2/DAP12 regulates the physiological functions of microglia from development to aging. Neurochem. Int. 141:104878. doi: 10.1016/J.NEUINT.2020.104878 33049336

[ref27] Lee C. K. Weindruch R. Prolla T. A. (2000). Gene-expression profile of the ageing brain in mice. Nat. Genet. 25, 294–297. doi: 10.1038/77046 10888876

[ref26] Lee J. Kim H. J. (2022). Normal aging induces changes in the brain and neurodegeneration Progress: review of the structural, biochemical, metabolic, cellular, and molecular changes. Front. Aging Neurosci. 14:931536. doi: 10.3389/FNAGI.2022.931536, PMID: 35847660 PMC9281621

[ref28] Liao H. Klaus C. Neumann H. (2020, 2020). Control of innate immunity by sialic acids in the nervous tissue. Int. J. Mol. Sci. 21:5494. doi: 10.3390/IJMS21155494 32752058 PMC7432451

[ref30] Liao H. Winkler J. Wißfeld J. Shahraz A. Klaus C. Neumann H. (2021). Low molecular weight polysialic acid prevents lipopolysaccharide-induced inflammatory dopaminergic neurodegeneration in humanized SIGLEC11 transgenic mice. Glia 69, 2845–2862. doi: 10.1002/GLIA.24073, PMID: 34406679

[ref29] Liao Y. Smyth G. K. Shi W. (2014). featureCounts: an efficient general purpose program for assigning sequence reads to genomic features. Bioinformatics 30, 923–930. doi: 10.1093/BIOINFORMATICS/BTT656, PMID: 24227677

[ref31] Love M. I. Huber W. Anders S. (2014). Moderated estimation of fold change and dispersion for RNA-seq data with DESeq2. Genome Biol. 15:550. doi: 10.1186/S13059-014-0550-8, PMID: 25516281 PMC4302049

[ref32] Luo X. G. Ding J. Q. Chen S. D. (2010). Microglia in the aging brain: relevance to neurodegeneration. Mol. Neurodegener. 5. doi: 10.1186/1750-1326-5-12, PMID: 20334662 PMC2852379

[ref33] Macauley M. S. Crocker P. R. Paulson J. C. (2014). Siglec-mediated regulation of immune cell function in disease. Nat. Rev. Immunol. 14, 653–666. doi: 10.1038/NRI3737, PMID: 25234143 PMC4191907

[ref34] Mootha V. K. Lindgren C. M. Eriksson K. F. Subramanian A. Sihag S. Lehar J.. (2003). PGC-1alpha-responsive genes involved in oxidative phosphorylation are coordinately downregulated in human diabetes. Nat. Genet. 34, 267–273. doi: 10.1038/NG1180, PMID: 12808457

[ref35] Mouton P. R. Long J. M. Lei D. L. Howard V. Jucker M. Calhoun M. E.. (2002). Age and gender effects on microglia and astrocyte numbers in brains of mice. Brain Res. 956, 30–35. doi: 10.1016/S0006-8993(02)03475-3, PMID: 12426043

[ref36] Mukherjee S. Klaus C. Pricop-Jeckstadt M. Miller J. A. Struebing F. L. (2019). A microglial signature directing human aging and neurodegeneration-related gene networks. Front. Neurosci. 13:2. doi: 10.3389/fnins.2019.00002, PMID: 30733664 PMC6353788

[ref37] Noda S. Sato S. Fukuda T. Tada N. Hattori N. (2020). Aging-related motor function and dopaminergic neuronal loss in C57BL/6 mice. Mol. Brain 13, 46. doi: 10.1186/S13041-020-00585-6, PMID: 32293495 PMC7092461

[ref38] Raj D. Yin Z. Breur M. Doorduin J. Holtman I. R. Olah M.. (2017). Increased white matter inflammation in aging- and Alzheimer’s disease brain. Front. Mol. Neurosci. 10:206. doi: 10.3389/FNMOL.2017.00206, PMID: 28713239 PMC5492660

[ref39] Rodrigue K. M. Kennedy K. M. (2011). “The cognitive consequences of structural changes to the aging brain” in Handbook of the psychology of aging, Eds. K. W. Schaie and S. L. Willis (Academic press) 73–91.

[ref390] RStudio Team. (2020). RStudio: Integrated development environment for R.RStudio. Available at: http://www.rstudio.com/

[ref40] Schwarz F. Pearce O. M. T. Wang X. Samraj A. N. Läubli H. Garcia J. O.. (2015). Siglec receptors impact mammalian lifespan by modulating oxidative stress. eLife 4:6184. doi: 10.7554/ELIFE.06184, PMID: 25846707 PMC4384638

[ref41] Shahraz A. Kopatz J. Mathy R. Kappler J. Winter D. Kapoor S.. (2015). Anti-inflammatory activity of low molecular weight polysialic acid on human macrophages. Sci. Rep. 5:16800. doi: 10.1038/srep16800, PMID: 26582367 PMC4652165

[ref9002] Shahraz A. Wißfeld J. Ginolhac A. Mathews M. Sinkkonen L. Neumann H. (2021). Phagocytosis-related NADPH oxidase 2 subunit gp91phox contributes to neurodegeneration after repeated systemic challenge with lipopolysaccharides. Glia, 69, 137–150. doi: 10.1002/GLIA.23890 32721081

[ref42] Shimabukuro M. K. Langhi L. G. P. Cordeiro I. Brito J. M. Batista C. M. D. C. Mattson M. P.. (2016). Lipid-laden cells differentially distributed in the aging brain are functionally active and correspond to distinct phenotypes. Sci. Rep. 6:3795. doi: 10.1038/SREP23795, PMID: 27029648 PMC4814830

[ref43] Subramanian A. Tamayo P. Mootha V. K. Mukherjee S. Ebert B. L. Gillette M. A.. (2005). Gene set enrichment analysis: a knowledge-based approach for interpreting genome-wide expression profiles. Proc. Natl. Acad. Sci. USA 102, 15545–15550. doi: 10.1073/PNAS.0506580102, PMID: 16199517 PMC1239896

[ref44] Tremblay M. È. Zettel M. L. Ison J. R. Allen P. D. Majewska A. K. (2012). Effects of aging and sensory loss on glial cells in mouse visual and auditory cortices. Glia 60, 541–558. doi: 10.1002/GLIA.22287, PMID: 22223464 PMC3276747

[ref45] Wang X. Chow R. Deng L. Anderson D. Weidner N. Godwin A. K.. (2011). Expression of Siglec-11 by human and chimpanzee ovarian stromal cells, with uniquely human ligands: implications for human ovarian physiology and pathology. Glycobiology 21, 1038–1048. doi: 10.1093/GLYCOB/CWR039, PMID: 21467073 PMC3130538

[ref46] Wang X. Mitra N. Cruz P. Deng L. Varki N. Angata T.. (2012). Evolution of Siglec-11 and Siglec-16 genes in hominins. Mol. Biol. Evol. 29, 2073–2086. doi: 10.1093/MOLBEV/MSS077, PMID: 22383531 PMC3408085

[ref47] Wang Y. Neumann H. (2010). Alleviation of neurotoxicity by microglial human Siglec-11. J. Neurosci. 30, 3482–3488. doi: 10.1523/JNEUROSCI.3940-09.2010, PMID: 20203208 PMC6634112

[ref48] Wickham H. (2016). ggplot2. Available at: 10.1007/978-3-319-24277-4 (Accessed July 8, 2024).

[ref49] Wißfeld J. Assale T. A. Cuevas-Rios G. Liao H. Neumann H. (2024). Therapeutic potential to target sialylation and SIGLECs in neurodegenerative and psychiatric diseases. Front. Neurol. 15:874. doi: 10.3389/FNEUR.2024.1330874, PMID: 38529039 PMC10961342

[ref50] Wong A. M. Patel N. V. Patel N. K. Wei M. Morgan T. E. De Beer M. C.. (2005). Macrosialin increases during normal brain aging are attenuated by caloric restriction. Neurosci. Lett. 390, 76–80. doi: 10.1016/J.NEULET.2005.07.058 16157452

[ref51] Xuan Q. Xu S. L. Lu D. H. Yu S. Zhou M. Uéda K.. (2011). Increased expression of α-synuclein in aged human brain associated with neuromelanin accumulation. J. Neural Transm. 118, 1575–1583. doi: 10.1007/S00702-011-0636-3, PMID: 21461961

[ref52] Yang W. Yu W. Li X. Li X. Yu S. (2020). Alpha-synuclein differentially reduces surface expression of N-methyl-d-aspartate receptors in the aging human brain. Neurobiol. Aging 90, 24–32. doi: 10.1016/J.NEUROBIOLAGING.2020.02.015, PMID: 32171588

[ref53] Yankner B. A. Lu T. Loerch P. (2008). The aging brain. Annu. Rev. Pathol. 3, 41–66. doi: 10.1146/ANNUREV.PATHMECHDIS.2.010506.092044 18039130

[ref54] Yu G. Wang L. G. Han Y. He Q. Y. (2012). clusterProfiler: an R package for comparing biological themes among gene clusters. Omics 16, 284–287. doi: 10.1089/OMI.2011.0118, PMID: 22455463 PMC3339379

