# Supplementary material for: Neuroprotective role of sialic-acid-binding immunoglobulin-like lectin-11 in humanized transgenic mice
Source: Front Neurosci. 2024 Dec 23;18:1504765. doi: 10.3389/fnins.2024.1504765 (PMC11701055; doi:10.3389/fnins.2024.1504765)
Supplement: Supplementary file 1 [file Data_Sheet_1.PDF]

Supplementary Material

Neuroprotective role of sialic-acid-binding immunoglobulin-like lectin-11 in transgenic mice

Tawfik Abou Assale*, Negin Afrang*, Jannis Wissfeld, German Cuevas-Rios, Christine Klaus, Bettina Linnartz-Gerlach* and Harald Neumann*

* equal contribution

Institute for Reconstructive Neurobiology, Medical Faculty and University Hospital of Bonn, University of Bonn, Venusberg-Campus 1, 53127 Bonn, Germany

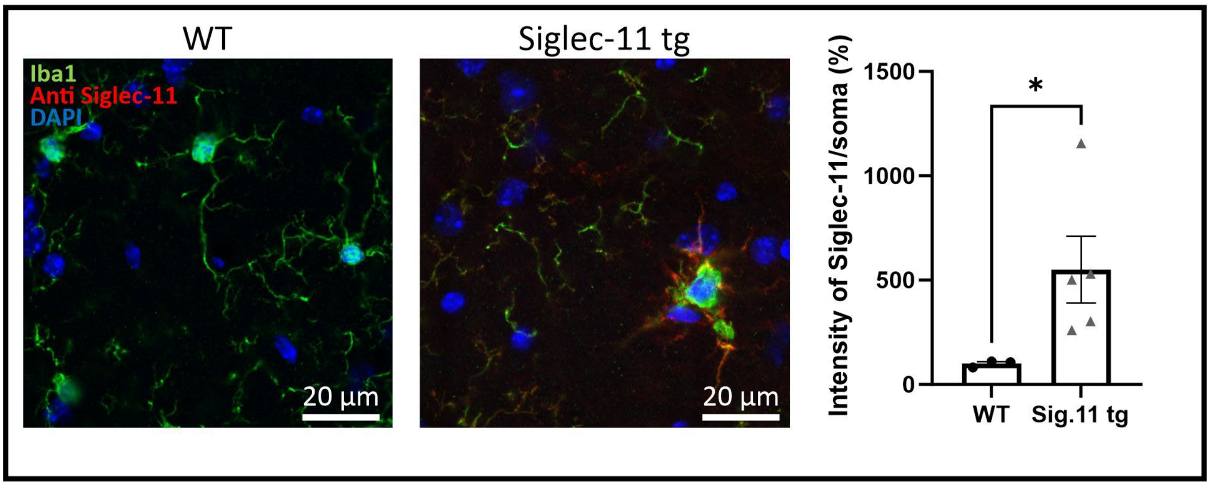

Supplementary fig. 1. Microglial SIGLEC-11 receptor expression in humanized Siglec-11 transgenic mice brain. (Left) Representative images of the microglial marker Iba1 (green), anti-Siglec-11 (red) and 4′,6-diamidino-2-phenylindole (DAPI, blue) in the brain sections of 24-month-old Siglec-11 transgenic (tg) and wildtype (WT) control mice. (Right) Quantification of relative SIGLEC-11 signal intensity per soma showed increased expression in Siglec-11 tg mice compared to WT control mice. Data analysed with Student's t-test and shown as mean±SEM; n= 3-5, **p* ≤ 0.05.

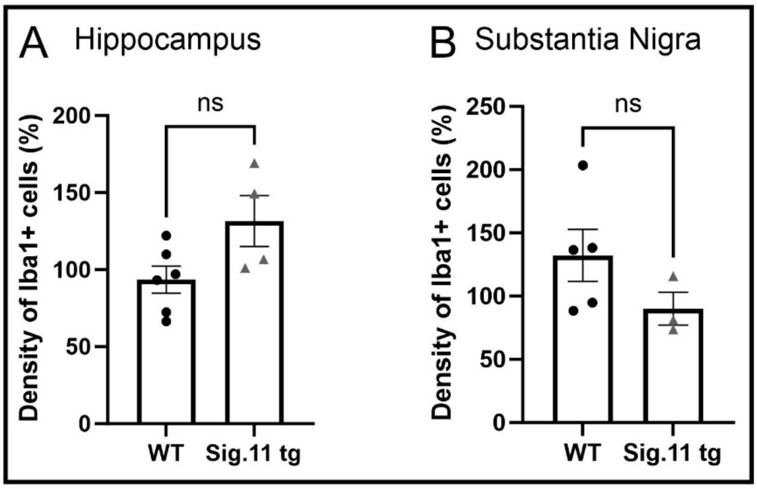

Supplementary fig. 2: **Microglial density quantification in the hippocampus and substantia nigra *pars reticulata* of Siglec-11 transgenic versus wildtype mice at 1.5 months of age.** Quantification of Iba1 cell density showed no significant difference between Siglec-11 transgenic (tg) mice and wildtype (WT) mice at 1.5 months of age in the **(A)** hippocampus and **(B)** substantia nigra. Data analysed with Student's t-test and shown as mean±SEM. n=3-6 mice per group. ns: not significant.

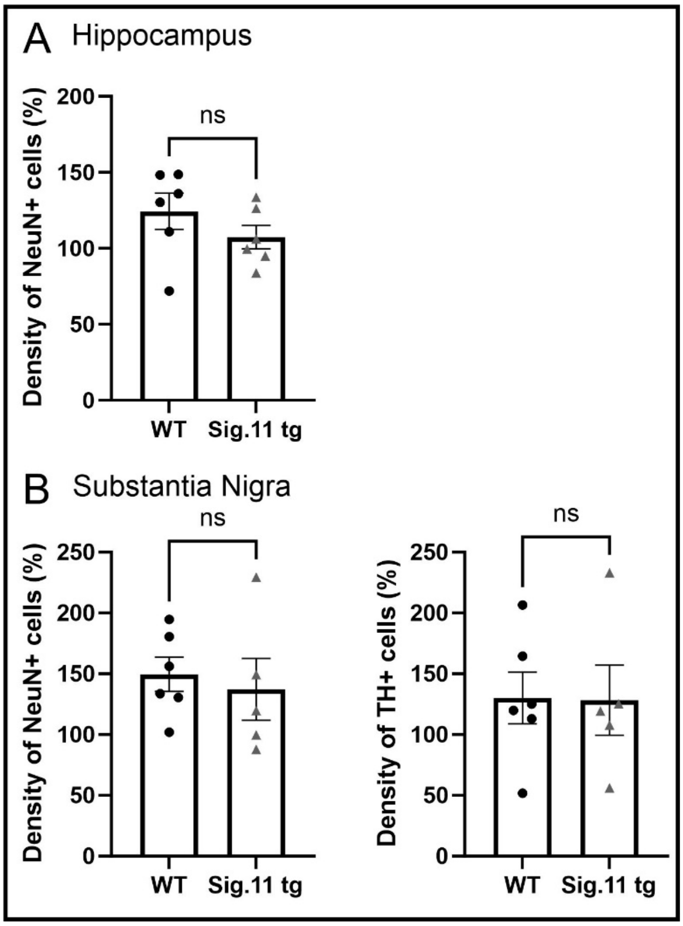

Supplementary fig. 3: Neuronal quantification in the hippocampus and substantia nigra of Siglec-11 transgenic versus wildtype mice at 1.5 months. (A) Quantification of NeuN-positive cell density in the hippocampal region showed no significant difference between wildtype (WT) and Siglec-11 transgenic (tg) mice at 1.5 months of age. **(B)** Quantification of NeuN-positive and TH-positive cell density in the substantia nigra *pars compacta* also showed no significant difference between Siglec-11 tg mice and WT mice at 1.5 months of age. Data analysed with Student's t-test and are shown as mean±SEM. n=5-6 mice per group. ns: not significant.

A

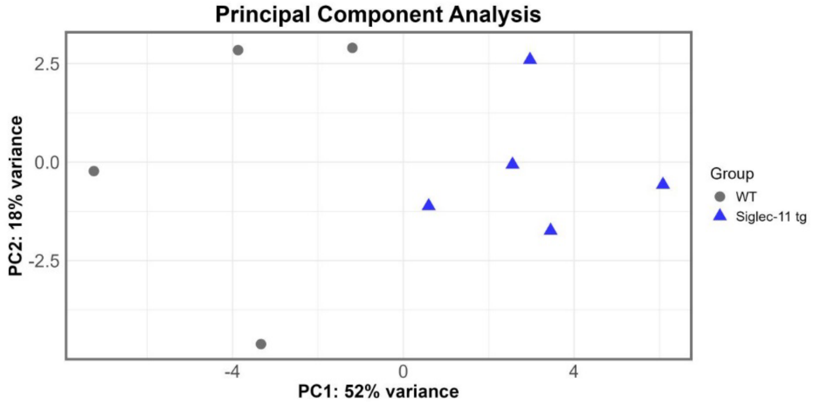

B

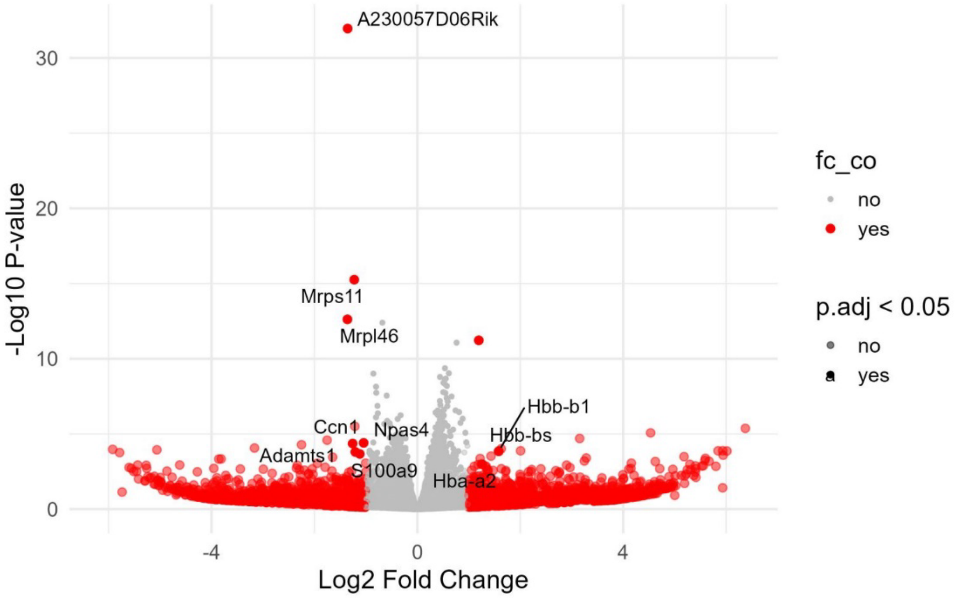

C

Hallmark Gene Set	NOM p-val	FDR Q-val
COAGULATION	0.0000	0.0695
APOPTOSIS	0.0000	0.1590
UV RESPONSE UP	0.0000	0.1389
XENOBIOTIC METABOLISM	0.0104	0.1147
NOTCH SIGNALING	0.0129	0.1309
CHOLESTEROL HOMEOSTASIS	0.0159	0.1042
OXIDATIVE PHOSPHORYLATION	0.0177	0.0200
EPITHELIAL MESENCHYMAL TRANSITION	0.0181	0.2231
TNFA SIGNALING VIA NFKB	0.0287	0.2078
REACTIVE OXIGEN SPECIES PATHWAY	0.0375	0.1367

Supplementary fig. 4. Transcriptomic analysis of 6 months old wildtype versus Siglec-11 transgenic mice. (A) Principal component analysis (PCA) of brain hemisphere transcriptomic RNA-sequencing analysis illustrating the multivariate distribution between 6-month-old Siglec-11 transgenic (tg; blue triangle) and wildtype (WT) control mice (grey dots). Each triangle/dot represents one individual mouse. **(B)** Volcano plot depicting the top differentially expressed genes of Siglec-11 tg mice compared to WT control mice. A p-value cut-off of less than 0.05 and log2FoldChange cut-off of greater than 1 were used to display the significantly differentially expressed genes. **(C)** Gene set enrichment analysis (GSEA) of the top 10 enriched hallmark gene sets sorted according to the nominal p-value revealed an enrichment in oxidative stress and apoptotic pathways in WT control mice in comparison to Siglec-11 tg mice. n=4-5 mice per group.

A

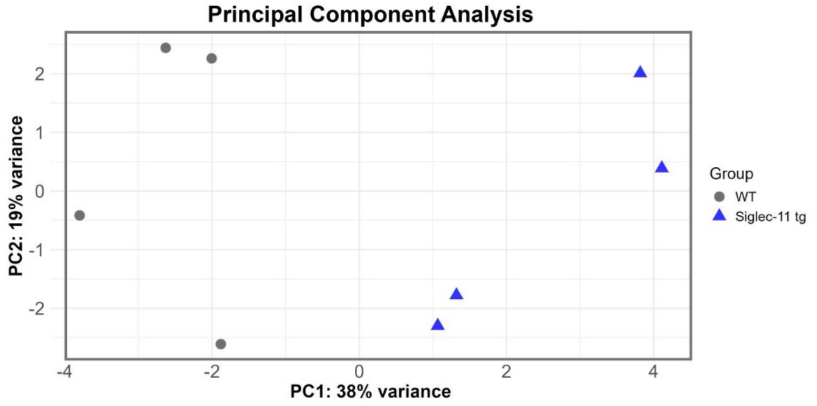

B

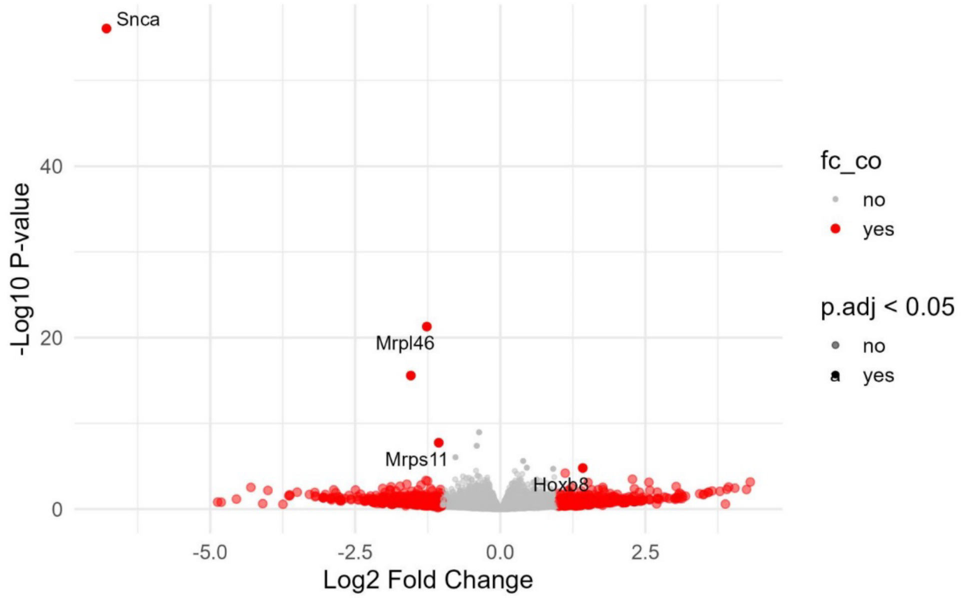

C

Hallmark Gene Set	NOM p-val	FDR Q-val
ANGIOGENESIS	0.0000	0.0328
KRAS SIGNALING UP	0.0000	0.2751
EPITHELIAL MESENCHYMAL TRANSITION	0.0353	0.3607
MTORC1 SIGNALING	0.0530	0.8074
COAGULATION	0.1095	0.8433
INFLAMMATORY RESPONSE	0.1699	0.7597
WNT BETA CATENIN SIGNALING	0.1905	0.8800
INTERFERON ALPHA RESPONSE	0.1975	0.7490
IL6 JAK STAT3 SIGNALING	0.2371	0.7794
COMPLEMENT	0.2400	0.7904

Supplementary fig. 5. Transcriptomic analysis of 24 months old wildtype versus Siglec-11 transgenic mice. (A) Principal component analysis (PCA) of brain hemisphere transcriptomic RNA-seq analysis illustrating the multivariate distribution between 24-month-old Siglec-11 transgenic (tg) mice (blue triangle) and WT control mice (grey dots). Each triangle/dot represents one individual mouse. **(B)** Volcano plot depicting the top differentially expressed genes of Siglec-11 tg mice compared to WT mice. A p-value cut-off of less than 0.05 and log2FoldChange cut-off of greater than 1 were used to display the significantly differentially expressed genes. **(C)** Gene set enrichment analysis (GSEA) of the top 10 enriched hallmark gene sets in WT mice sorted according to the nominal p-value showed enrichment in complement, apoptotic and epithelial mesenchymal transition gene sets in comparison to Siglec-11 tg mice. n=4 mice per group.

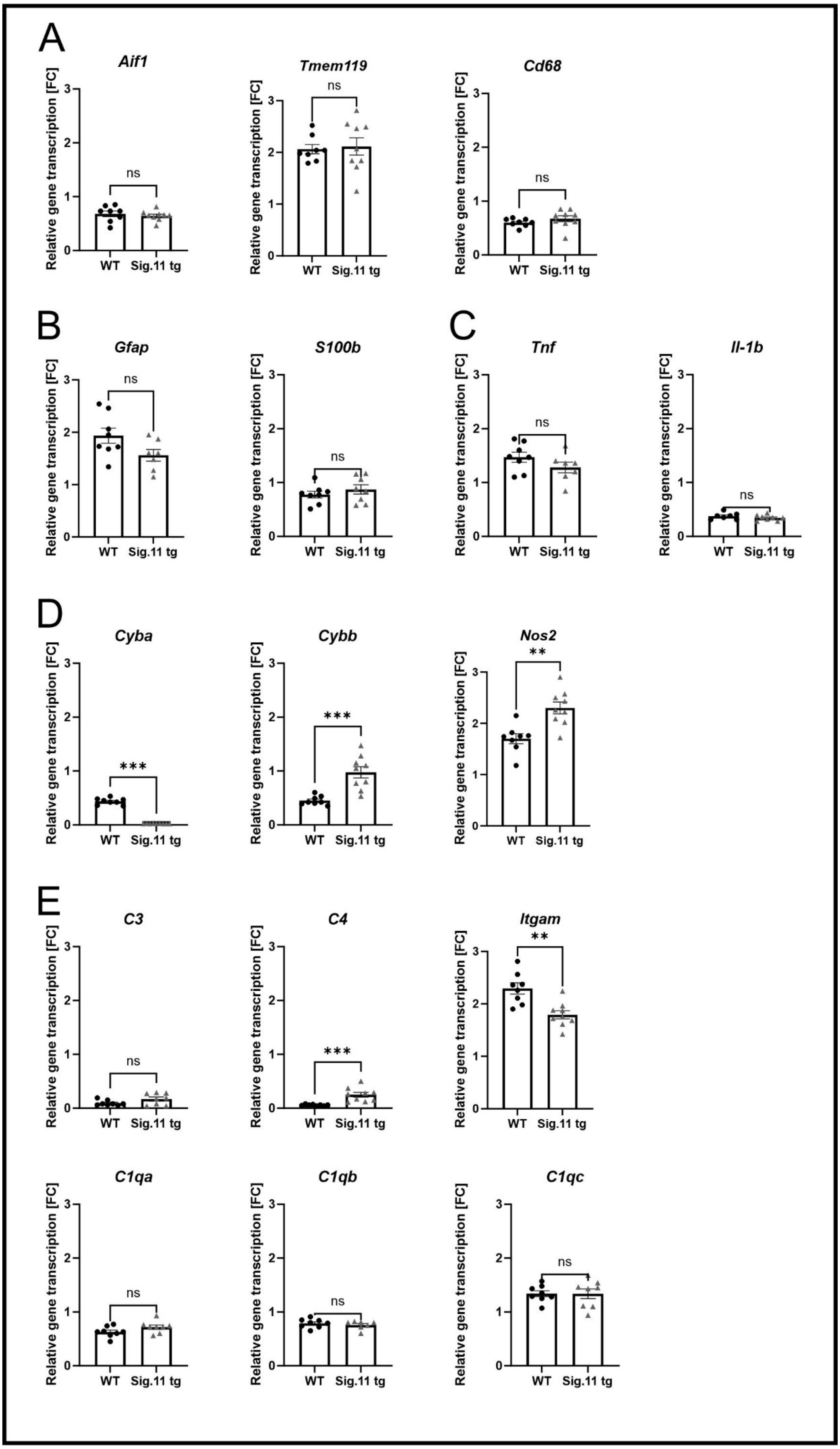
A
Aif1
Tmem119
Cd68
B
Gfap
S100b
C
Tnf
Il-1b
D
Cyba
Cybb
Nos2
E
C3
C4
Itgam
C1qa
C1qb
C1qc
Relative gene transcription [FC]
WT
Sig.11 tg
ns
**
***

Supplementary fig. 6. Semi-quantitative real-time PCR (sqRT-PCR) analysis of microglial and astrocytic markers, along with pro-inflammatory cytokines, oxidative stress markers and complement components in brain hemisphere homogenates of wildtype versus Siglec-11 transgenic mice at 1.5 months of age. Transcription of the **(A)** microglial markers *Aif1* (allograft inflammatory factor 1), *Tmem119* (transmembrane protein 119), and *Cd68*, **(B)** astrocytic markers *gfap* (glial fibrillary acidic protein) and *S100b* (S100 calcium-binding protein B), and **(C)** pro-inflammatory cytokines *Tnf* (tumor necrosis factor-α) and *Il-1β* (interleukin-1β) showed no difference between Siglec-11 transgenic (tg) and wildtype (WT) mice at 1.5 months of age. **(D)** The oxidative stress markers showed a decreased transcription of *Cyba* (cytochrome b-245 alpha) in the Siglec-11 tg mice compared to WT mice, while *Cybb* (cytochrome b-245 beta) and *Nos2* (nitric oxide synthase 2) transcription was elevated in Siglec-11 tg mice in comparison to WT mice. **(E)** Complement component factors showed increased transcription of the *C4* (complement component 4) gene in Siglec-11 tg mice in comparison to WT mice, while the transcription of *Itgam* (integrin alpha M) was decreased in Siglec-11 tg mice compared to WT mice. Data analyzed with Student's t-test and are shown as mean±SEM. n=6-9 mice per group. ns: not significant, **$p \leq 0.01$, ***$p \leq 0.001$.

Supplementary Table 1. Oligonucleotides

Target	Orientation	Sequence (5’-3’)
Aif-1	forward	GAAGCGAATGCTGGAGAAAC
	reverse	AAGATGGCAGATCTCTTGCC
C1qA	forward	AGAGGGGAGCCAGGAGC
	reverse	CTTTCACGCCCTTCAGTCCT
C1qB	forward	GACTTCCGCTTTCTGAGGACA
	reverse	CAGGGGCTTCCTGTGTATGGA
C1qC	forward	GCCTGAAGTCCCTTACACCC
	reverse	GGGATTCCTGGCTCTCCCTT
C3	forward	TAGTGCTACTGCTGCTGTTGGC
	reverse	GCTGGAATCTTGATGGAGACGCTT
C4	forward	TGGAGGACAAGGACGGCTA
	reverse	GGCCCTAACCCTGAGCTGA
Cd68	forward	CAGGGAGGTTGTGACGGTAC
	reverse	GAAACATGGCCCGAAGTATC
Cyba	forward	CCTCCACTTCCTGTTGTCGG
	reverse	TCACTCGGCTTCTTTCGGAC
Cybb	forward	GGGAACTGGGCTGTGAATGA
	reverse	CAGTGCTGACCCAAGGAGTT
Gapdh	forward	ACAACTTTGGCATTGTGGAA
	reverse	GATGCAGGGATGATGTTCTG
Gfap	forward	CACGTGGAGATGGATGTGGC
	reverse	CTCCAGATCGCAGGTCAAGG
Il1β	forward	CTTCCTTGTGCAAGTGTCTG
	reverse	CAGGTCATTCTCATCACTGTC
Itgam	forward	CATCAAGGGCAGCCAGATTG
	reverse	GAGGCAAGGGACACACTGAC
Nos2	forward	AAGCCCCGCTACTACTCCAT
	reverse	GCTTCAGGTTCCTGATCCAA
S100b	forward	GTCTTCCACCAGTACTCCGG
	reverse	ACGAAGGCCATGAACTCCTG
Siglec-11 genotyping	forward	GGAGATGTCAGGGATGGTTC
	reverse	AGCAGCGTATCCACATAGCGT
Tmem119	forward	GTGTCTAACAGGCCCCAGAA
	reverse	AGCCACGTGGTATCAAGGAG
Tnf	forward	GGTGCCTATGTCTCAGCCTC
	reverse	TGAGGGTCTGGGCCATAGAA
